# Diallyl Trisulfide Induces ROS-Mediated Mitotic Arrest and Apoptosis and Inhibits HNSCC Tumor Growth and Cancer Stemness

**DOI:** 10.3390/cancers16020378

**Published:** 2024-01-16

**Authors:** Sivapar V. Mathan, Ragini Singh, Su-Hyeong Kim, Shivendra V. Singh, Rana P. Singh

**Affiliations:** 1 Cancer Biology Laboratory, School of Life Sciences, Jawaharlal Nehru University, New Delhi 110067, India; sivaparmathan@gmail.com (S.V.M.); raginisingh17nov@gmail.com (R.S.); 2 Department of Pharmacology and Chemical Biology, University of Pittsburgh School of Medicine, Pittsburgh, PA 15213, USA; kims7@upmc.edu (S.-H.K.); svs2@pitt.edu (S.V.S.); 3 UPMC Hillman Cancer Center, University of Pittsburgh School of Medicine, Pittsburgh, PA 15213, USA; 4 Special Centre for Systems Medicine, Jawaharlal Nehru University, New Delhi 110067, India

**Keywords:** diallyl trisulfide, HNSCC, mitotic arrest, apoptosis, DNA damage, cancer stem cells

## Abstract

**Simple Summary:**

Head and neck cancers arise in the mouth, pharynx, salivary glands, and larynx, and most of these cancers are squamous cell carcinomas. Causative agents for head and neck squamous cell carcinoma (HNSCC) include tobacco-derived carcinogens, alcohol consumption, and HPV infection. Currently, the survival rate is poor, and available chemotherapeutic agents are associated with toxicities and chemoresistance largely due to cancer stem cells (CSC). Therefore, the development of agents that are nontoxic to normal cells, and inhibit the growth of both head and neck cancer cells as well as CSC are desired. Epidemiological studies showed that dietary intake of Allium vegetables lowered the risk of various cancers, including head and neck cancer. Several studies have reported processed-garlic-constituent diallyl trisulfide (DATS) as a promising compound that induced growth arrest and apoptosis in prostate and breast cancer. In the present study, DATS decreased cell viability, and induced growth arrest and apoptotic cell death involving DNA damage and reactive oxygen species generation in HNSCC cells. DATS also reduced CD133^high^/CD44^high^ CSC fraction, spheroid formation and aldehyde dehydrogenase 1 (ALDH1) activity, and downregulated *Oct4* and *SOX2* expression. Further, DATS inhibited HNSCC tumor growth and CSC fraction in the tumor xenograft model. Thus, DATS could be a potential anticancer agent against head and neck cancer.

**Abstract:**

Despite advances in therapeutic approaches, the five-year survival rate for head and neck squamous cell carcinoma (HNSCC) patients is still less than fifty percent. Research has indicated that the consumption of Allium vegetables or processed garlic containing diallyl trisulfide (DATS) can lower the risk of multiple types of cancer. Nevertheless, the effectiveness and underlying mechanisms of DATS against HNSCC have not been thoroughly explored until the current study. In this research, it was found that DATS notably curtailed the growth and viability of HNSCC cells. Additionally, DATS triggered a significant G2/M cell cycle arrest in these cells, accumulating cyclin B1, Cip1/p21, and Ser-10 phospho-histone H3—this was indicative of mitotic arrest attenuated by NAC pretreatment, suggesting the role of reactive oxygen species (ROS) induction. The production of ROS induced by DATS led to DNA damage and apoptosis, a process associated with elevated levels of cleaved caspase-3 and cleaved PARP, along with reduced XIAP. When HNSCC cells were exposed to pharmacological concentrations of DATS, it resulted in the suppression of cancer stem cell (CSC) populations, as indicated by a decrease in the CD133^high^/CD44^high^ cell fraction, reduced aldehyde dehydrogenase 1 (ALDH1) activity, inhibited spheroid formation and downregulated *SOX2* and *Oct4* expression. Furthermore, the administration of DATS to tumor xenografts demonstrated its in vivo capacity to hinder CSCs. Further, DATS treatment inhibited the growth of UMSCC-22B head and neck cancer tumor xenograft in immunocompromised mice. Overall, DATS inhibited cell proliferation; induced cell cycle mitotic arrest and apoptosis involving DNA damage through ROS generation; reduced the CSC fraction and spheroid formation; and downregulated *SOX2* and *Oct4* expression. More importantly, DATS inhibited HNSCC tumor growth and CSC fraction in vivo. Thus, DATS could be a potential anticancer agent that can be used against head and neck cancer.

## 1. Introduction

Head and neck cancer arises in the oral cavity, pharynx, and larynx. These cancers have >90% squamous cell histology and, thus, are generally referred to as head and neck squamous cell carcinoma (HNSCC) [1]. Head and neck cancer is the seventh most common cancer prevalent worldwide and the leading common cancer in men in India. Despite significant advances in surgery, chemoradiotherapy, and targeted molecular approaches, the five-year survival rate is very poor [2]. Although head and neck cancer are curable during the early stages, unfortunately more than 60% of patients are diagnosed in advanced stages. Early detection and treatment could help in downstaging and preventing invasive cancer from spreading, thereby improving the survival rates of HNSCC. Nevertheless, chemoprevention may serve as a better strategy for managing HNSCC and benefitting those at high risk of cancer recurrence [3,4,5].

A potential cause of tumor relapse might be that conventional therapies only target cancer cells, but miss slow-growing cancer stem cells (CSC) or tumor-initiating cells (TICs) that can repopulate the tumor and result in tumor recurrence [6]. Studies have shown that cisplatin, the most commonly used chemotherapeutic drug for HNSCC, enhanced CSC fraction [7]. Therefore, CSCs have become an attractive target for the development of novel therapeutic strategies. Studies have shown that phytochemicals that have antioxidative properties have anticancer effects and are shown to target CSCs [6]. Thus, a tremendous unmet need remains for an effective agent that targets both bulk cells of head and neck cancer cells as well as CSCs.

The medicinal benefits of *Allium* species have been well known since ancient times. Epidemiological studies have shown an inverse correlation between the intake of Allium vegetables and the risk of different types of malignancies, including HNSCC. Allium vegetable intake reduced the risk of esophageal [8], stomach [9], pancreatic [10], prostate [11], endometrial [12], and colorectal cancers [13]. Research suggests that the cancer-fighting properties of Allium vegetables are linked to the presence of organosulfur compounds (OSCs), which are released when these vegetables are cut or chewed. OSCs derived from Allium vegetables, such as diallyl sulfide (DAS), diallyl disulfide (DADS), and diallyl trisulfide (DATS), have demonstrated substantial protective effects against cancer in animal models induced by various chemical carcinogens [14]. Anticancer effects of DATS were shown in prostate [15], breast [16], and lung cancer [17]. However, the efficacy and molecular mechanisms of its chemopreventive effects against HNSCC have not been investigated.

In the present study, we investigated the effect of DATS on the growth, proliferation, and survival of UMSCC-22A, UMSCC22-B, and Cal33 HNSCC cells. Its effect on cell cycle progression and mitotic arrest, DNA damage, apoptosis, and associated markers and mechanisms, including the role of reactive oxygen species (ROS) production, was also studied. Further, DATS effect on CSCs and spheroid formation, followed by its in vivo effects on HNSCC tumor xenograft growth and fraction of CSCs in tumors, were also studied.

## 2. Material and Methods

### 2.1. Reagents and Cell Lines

Cell culture essentials, including DMEM (Dulbecco’s Modified Eagle Medium), sodium pyruvate, non-essential amino acids, fetal bovine serum (FBS), and penicillin/streptomycin antibiotic mixture, were obtained from GIBCO (Grand Island, NY, USA). Diallyl trisulfide (DATS, purity > 98%) was procured from LKT Laboratories (St. Paul, MN, USA), and RNase A was sourced from Promega (Madison, WI, USA). Cyclin B1, Cdk1, pERK1/2, ERK1 antibodies were purchased from Santa Cruz Biotechnology (Santa Cruz, CA, USA); the anti-beta actin antibody was purchased from Sigma (St. Louis, MO, USA); and the antibody against cleaved poly-(ADP-ribose)-polymerase (c-PARP), cleaved caspase-3, pAkt (Ser 473), Akt, γH2AX (Ser 139), were purchased from Cell Signaling Technology (Danvers, MA, USA). UMSCC-22A, UMSCC-22B, and Cal33 cells were a generous gift from Prof. Daniel E. Johnson (University of California, San Francisco, CA, USA). HNSCC cells were grown in DMEM and supplemented with 10% FBS, 100 µg/mL streptomycin, and 100 units/mL penicillin under standard culture conditions. Stock solution of DATS was prepared in dimethyl sulfoxide (DMSO).

### 2.2. Cell Viability Assay

Cells were seeded and allowed to adhere overnight, and treated with fresh medium containing different concentrations of DATS, and the plates were incubated for 24, 48, or 72 h at 37 °C. After incubation, cells were harvested and processed, and counted with 0.4% trypan blue solution, as previously detailed [18].

### 2.3. Cell Cycle Analysis

Briefly, cells were treated with the desired concentrations of DATS. After incubation, cells were harvested, processed, and stained with propidium iodide, as described earlier. Stained cells were analyzed by flow cytometry, using the BD FACS Aria flow cytometer (BD Biosciences, Franklin Lakes, NJ, USA) [19].

### 2.4. Annexin V FITC Apoptosis Assay

For quantitation of apoptosis by flow cytometry using Annexin V/Propidium Iodide Apoptosis Detection kit (BD Biosciences, Franklin Lakes, NJ, USA), cells were treated with DMSO or DATS for 24 h. Cells were collected, processed, and stained following the manufacturer’s protocol. Stained cells were analyzed using a BD Accuri^TM^ C6 flow cytometer (BD Biosciences). Similarly, for NAC pretreatment apoptotic assay, stained cells were analyzed using FACS Aria III (BD Biosciences) [20].

### 2.5. Flow Cytometric Analysis of Mitotic Marker Phospho-(Ser10)-Histone H3

Cells were treated with DATS, as described above, and harvested, processed and incubated with anti-phospho-(Ser10)-histone H3 antibody, as described earlier [21]; cellular fluorescence was measured by BD Accuri^TM^ C6 flow cytometer (BD Biosciences). Similarly, this experiment was repeated with the pretreatment of 4 mM antioxidant, N-acetyl cysteine (NAC).

### 2.6. Detection of ROS by DCFDA Assay

To measure ROS generation, we initially seeded 10,000 cells per well in 96-well plates. After a 24-h incubation period, cells were incubated for 30 min with 10 µM DCFHDA. Subsequently, we removed the DCFHDA solution and performed two washes with serum-free media. Prior to treating the cells with DATS, we subjected certain wells to a 2-h pre-treatment with 4 mM NAC to inhibit ROS formation. Finally, we utilized a multi-plate reader with an excitation/emission wavelength of 485/538 nm to measure fluorescence [22].

### 2.7. Reactive Oxygen Species Staining by MitoSOX

Cells were treated for 3 h with DMSO and desired concentrations of DATS, and then incubated with 5 µM MitoSOX Red for 30 min. Cells were collected and washed with PBS, and fluorescence was detected using a BD Accuri^TM^ C6 flow cytometer (BD Biosciences) [23].

### 2.8. Comet Assay

The Comet assay was conducted following the previously described procedure [24]. After the desired treatment, cells were harvested and mixed with 1% low melting point agarose. This mixture was then applied to a microscope slide’s surface and immersed in a lysis buffer for a duration of 4 h at 4 °C, and then in an unwinding buffer for 20 min at 4 °C. Subsequently, the slides were immersed in neutralization buffer for an additional 20 min. This was followed by gel electrophoresis, and staining with ethidium bromide. Cells were visualized by fluorescence microscope and comet tail length was analyzed, as described previously [24].

### 2.9. Western Blot Analysis

As previously detailed, cells were treated with varying concentrations of DATS for 24 h and then harvested. Whole-cell lysate preparation, protein quantification, and western blotting were performed, as described previously [25].

### 2.10. Flow Cytometric Analysis of CD44 and CD133 Cancer Stem Cell Markers

Cells were similarly seeded and treated with DMSO and desired concentrations of DATS, as in the flow cytometric cell cycle analysis mentioned above. For flow cytometric analysis of CD133^high^ and CD44^high^, DATS-treated cells were incubated with anti-CD133 and anti-CD44 antibodies. Cells were incubated in the dark for 30 min at room temperature, followed by washing with PBS and then analyzed by using a BD Accuri C6 flow cytometer [26].

### 2.11. Aldehyde Dehydrogenase Assay

The flow cytometric quantitation of ALDH1-positive cells was carried out by following the manufacturer’s instructions (Stem Cell Technologies, Vancouver, BC, Canada). Briefly, after trypsinization followed by PBS wash, cells were resuspended with 600 µL of ALDEFLUOR assay buffer containing an ALDH1 substrate (bodipy-aminoacetaldehyde, BAAA), before half of a sample was transferred to a new tube containing the ALDH1 inhibitor diethylaminobenzaldehyde (DEAB), as a positive control. Then, the cells were incubated at 37 °C in an incubator for 30 min in the dark. After incubation, the cells were washed with PBS twice, and pelleted cells were resuspended with 200 µL ALDEFLUOR assay, including 1 µg/mL of PI, before BD Accuri C6 Flow Cytometer analysis proceeded [26].

### 2.12. Spheroid Formation Assay

The culture medium was aspirated out from the culture plate and cells were rinsed with warm PBS or HBSS, followed by trypsinization and pipetting for single cells. Cells were centrifuged and the supernatant was discarded. The pellet was suspended, and pipetting was done to obtain single cells and further sieved through a 40 μm sieve to obtain single cells. These single cells were plated in ultra-low attachment plates (Corning, Corning, NY, USA) at a density of 1000 cells/well in serum-free low glucose DMEM media containing 1% penicillin/streptomycin, B-27 (Gibco, Grand Island, NY, USA), insulin (Santa Cruz, Santa Cruz, CA, USA), hydrocortisone (Sigma, St. Louis, CA, USA), EGF (R&D systems, Minneapolis, MN, USA), bFGF (Stem Cell) and β-mercaptoethanol. Desired concentrations of DATS were added to the media in the primary sphere formation assay. Spheres were counted in an inverted microscope after 14 days [26].

### 2.13. RNA Extraction and Quantitative Real-Time PCR

Cellular total RNA extraction was executed by employing the TRIzol reagent from Takara Biosciences (San Jose, CA, USA). RNA was reverse-transcribed into complementary DNA (cDNA) synthesis and the quantitative real-time polymerase chain reaction (qRT-PCR) reactions were performed, as described previously [27]. Primers were as follows; *Oct-4*: forward 5′-GACAACAATGAAAATCTTCAGGAGA-3′, reverse 5′-CTGGCGCCGGTTACAGAACCA-3′; *SOX-2*, forward 5′-GCATGGACAGTTACGCGCAC-3′, reverse 5′-GCCGTTCATGTAGGTCTGCG-3′; *GAPDH*, forward 5′-CCCCTTCATTGACCTCAACTACA-3′, reverse 5′-TGACAAGCTTCCCGTTCTCA-3′. Relative gene expression levels were calculated using the method of Livak and Schmittgen [28].

### 2.14. Tumor Xenograft Study

Six-week-old female nude mice were purchased from The Jackson Laboratory (Bar Harbor, ME, USA), and were injected subcutaneously on both right and left flanks with exponentially growing UMSCC-22B. The mice were categorized into two groups, each consisting of five mice. One group received treatment through oral gavage, with the administration of a vehicle, while the other group received 2 mg/Kg of DATS via oral gavage three times a week for two weeks.

In another study, six-week-old nude mice were injected subcutaneously on both right and left flanks with exponentially growing UMSCC-22B cells. The mice were divided into two groups of four mice per group. On the 3rd day, treatment by oral gavage with either vehicle or 2 mg/Kg of DATS was performed five times each week for two weeks. Diet and water consumption, tumor size/volume, and weights were recorded routinely, as described previously [26]. Immunohistochemistry (IHC) staining and analysis were done, as described previously [29]. At least five nonoverlapping representative images were captured from each section and analyzed using Aperio ImageScope v9.1 software.

### 2.15. Statistical and Densitometry Analyses

Statistical analysis of the data was performed using GraphPad Prism 9 software. All the results of in vitro experiments were reproducible in two-three independent experiments. The significance of differences among control and treatment groups was assessed using an unpaired two-tailed Student’s *t*-test, with a significance threshold set at *p* < 0.05. For multiple comparisons, a one-way ANOVA test was employed. Band intensities were quantified using UN-SCAN-IT 7.1 and were presented as fold changes beneath each corresponding band.

## 3. Results

### 3.1. DATS Strongly Inhibited the Growth and Proliferation of Head and Neck Cancer Cells and Induced G2/M Phase Cell Cycle Arrest

**Figure 1:**
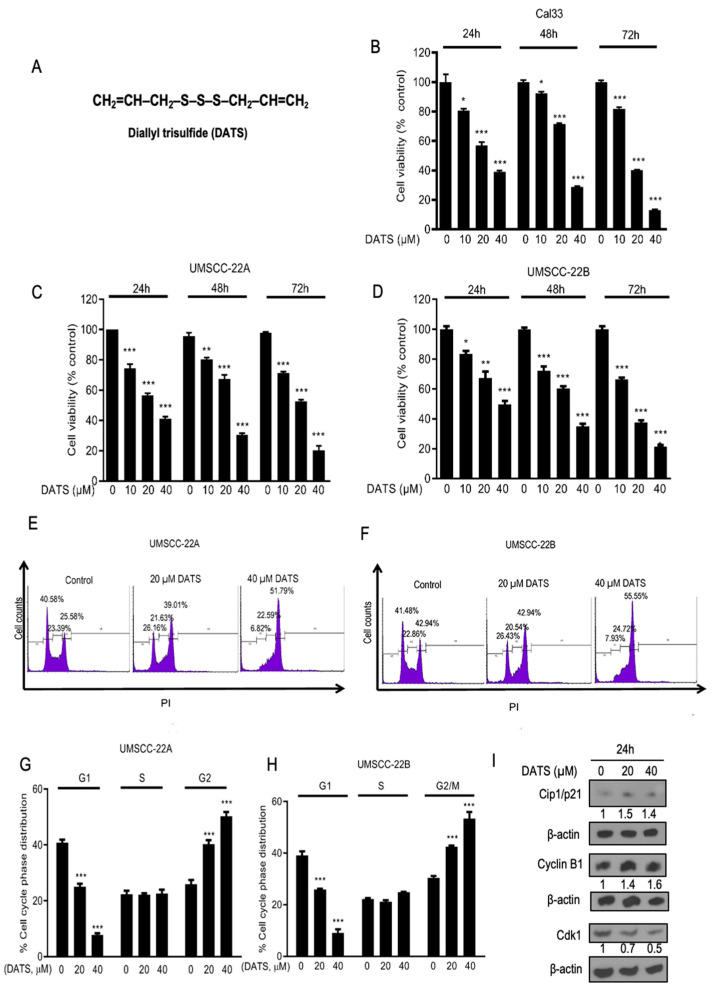
DATS inhibits cell viability and induces cell cycle arrest in head and neck cancer cells. (**A**) Structure of DATS. Effect of DATS on Cal33 (**B**), UMSCC-22A (**C**) and UMSCC-22B (**D**). Cells were treated with vehicle (DMSO) alone or 10–40 µM of DATS in a fresh medium. After 24, 48, and 72 h of these treatments, viable cells were counted using trypan blue staining and hemocytometer. DATS treatment caused G2/M phase cell cycle arrest in HNSCC cells. Representative flow histograms depicting cell cycle distribution in UMSCC-22A (**E**) and UMSCC-22B (**F**) cell cultures following 8 h treatment with the indicated concentrations of DATS. Quantitative analysis of UMSCC-22A (**G**) and UMSCC-22B (**H**) cells in different phases of the cell cycle after treatment with indicated concentrations of DATS. (**I**) Western blotting for cell cycle regulatory proteins using lysate from UMSCC-22B cells treated with DMSO control and DATS (20, 40 μM) for 24 h. The results shown are mean ± SEM (*n* = 3). DATS, Diallyl trisulfide, *p* < 0.05 (*), *p* < 0.001 (**), *p* < 0.0001 (***). The original western blot of Figure 1I is in Figure S6.

DATS (Figure 1A) treatments using 10–40 µM doses to Cal33 cells induced growth inhibition by 19.5% to 60.9% (*p* < 0.001) at 24 h, 7.8% to 71.2% (*p* < 0.0001) at 48 h and 18.1% to 86.8% (*p* < 0.0001) at 72 h (Figure 1B). DATS treatments (10–40 µM) to UMSCC-22A cells induced growth inhibition by 25.5% to 58.8% (*p* < 0.0001) at 24 h, 15.4% to 65.2% (*p* < 0.05–0.0001) at 48 h and 26.5% to 77.6% (*p* < 0.0001) at 72 h (Figure 1C) and in the case of UMSCC-22B cells, growth inhibition was 16.4% to 50.3% (*p* < 0.05–0.001) at 24 h, 27.7% to 64.9% (*p* < 0.0001) at 48 h and 33.5% to 78.5% (*p* < 0.0001) at 72 h (Figure 1D). DATS treatment moderately induced growth inhibition in HEK 293 cells at 24 h (Supplementary Figure S1). The cell cycle phase distribution of UMSCC-22A and UMSCC-22B cells after 24 h exposure to DMSO, 20 µM, and 40 µM DATS analysis was performed (Figure 1E,F). Exposure of HNSCC cells to growth-inhibitory concentrations of DATS resulted in a statistically significant increase in the G2/M fraction of cells. DATS treatments specifically arrested UMSCC-22A and UMSCC-22B cells at the G2/M phase of the cell cycle under normal growth conditions. Compared to 25.9% cells in the G2/M phase in DMSO control, DATS treatments employing 20 and 40 µM doses in UMSCC-22A cells increased the G2/M cell population up to 40.2% to 50.2% (*p* < 0.0001) at 24 h (Figure 1G). Similarly, after administering DATS treatments to UMSCC-22B cells, we observed that, compared to 30.3% of cells in the G2/M phase in control, the G2/M cell population increased up to 42.4%, to 53.4% (*p* < 0.0001) at 24 h (Figure 1H). These observations suggested that DATS conducts anticancer activities against HNSCC cells and inhibits the growth of cancer cells by inducing G2/M phase cell cycle arrest. Further, we studied the effect of DATS on cell cycle regulatory proteins: DATS-mediated G2/M phase cell cycle arrest in UMSCC-22B cells was associated with a decrease in the level of Cdk1 protein; an increase in cell cycle inhibitor Cip1/p21; and accumulation of cyclin B1 protein level (Figure 1I). Similarly, DATS treatment induced the accumulation of cyclin B1 and increased Cip1/p21 level in UMSCC-22A cells (Supplementary Figure S2). We further checked if cells were arrested in the G2 phase and/or M phase.

### 3.2. DATS Induced ROS-Mediated Mitotic Arrest and Altered G2/M Regulatory Proteins in HNSCC Cells

**Figure 2:**
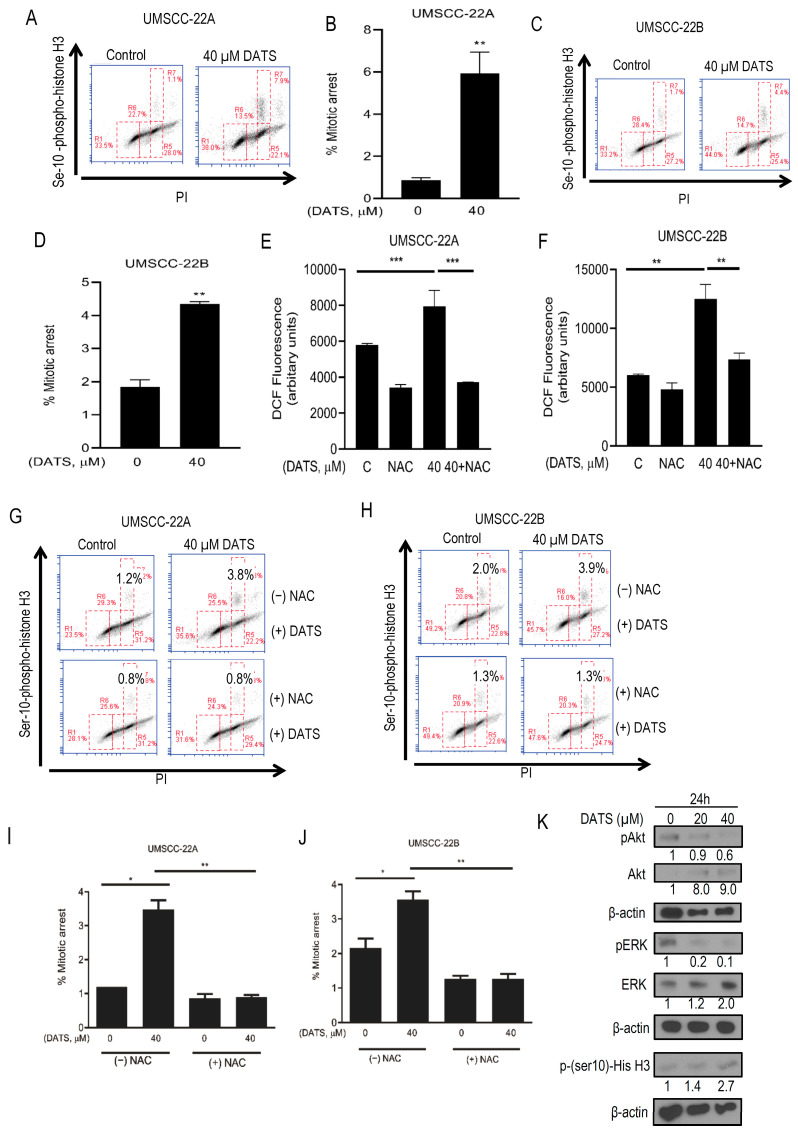
DATS treatment caused ROS-mediated mitotic arrest and altered cell cycle regulatory proteins in HNSCC cells. Representative flow histogram depicting Ser-10 phosphorylated histone H3 in UMSCC-22A (**A**) and UMSCC-22B (**C**) cells treated for 24 h with DMSO (control) or 40 μM DATS. Quantitation of the percentage of mitotic fraction in UMSCC-22A (**B**) and UMSCC-22B (**D**) cells treated with DMSO (control) or 40 μM DATS. DCFDA assay for ROS generation in DATS (40 μM) and/or NAC (4 mM) treated UMSCC-22A (**E**) and UMSCC-22B (**F**) cells. Representative histograms depicting Ser-10 phosphorylated histone H3 in UMSCC-22A (**G**) and UMSCC-22B (**H**) cells treated for 24 h with DMSO (control) or 40 μM DATS in the absence or presence of 4 mM NAC pretreatment. Quantified data of percent mitotic arrest in UMSCC-22A (**I**) and UMSCC-22B (**J**) cells upon DATS and/or NAC treatment. (**K**) Western blotting for cell proliferation and survival proteins using lysate from UMSCC-22B cells treated with DMSO control and DATS (20, 40 μM) for 24 h. Data are shown as mean ± SEM of triplicate samples. DATS, Diallyl trisulfide, *p* < 0.05 (*), *p* < 0.001 (**), *p* < 0.0001 (***). The original western blot of Figure 2K is in Figure S6.

DATS-induced mitotic arrest in HNSCC cells was confirmed by flow cytometric analysis of Ser10 phosphorylation of histone H3, a sensitive marker for mitotic cells [30]. The Ser10 phosphorylation of histone H3 was increased by about 6.8-fold and 2.3-fold (*p* < 0.05) upon treatment of UMSCC-22A and UMSCC-22B cells with 40 µM DATS at 24 h, compared with control, respectively (Figure 2A–D). These results revealed that DATS-treated UMSCC-22A and UMSCC-22B cells were arrested in the mitotic M phase.

Studies have evaluated the pro-oxidant effect of several chemopreventive agents, including DATS in cancer cells, and have demonstrated that their biological effects could be attributable to enhanced intracellular ROS levels [31]. To study the effect of DATS on ROS generation, cells were exposed to DATS and the changes in DCF fluorescence were measured. DCFDA assay showed a significant increase in intracellular ROS level in DATS-treated cells and NAC pretreatment significantly attenuated the DATS-induced ROS generation in both UMSCC-22A and UMSCC-22B cells (Figure 2E,F). In addition, we used a chemical probe for ROS detection (i.e., MitoSOX Red. DATS (20 and 40 µM)); treatment to UMSCC-22A and UMSCC-22B cells showed a 1.9-fold (*p* < 0.05–0.0001) increase in ROS production in UMSCC-22A cells and a 2.2-fold (*p* < 0.05–0.0001) increase in UMSCC-22B cells (Supplementary Figure S3A,B). We further checked if ROS generation has a role in DATS-induced mitotic arrest: cells were treated with 4 mM of NAC for 2 h, followed by 40 µM DATS treatment for 24 h, and mitotic phase cell cycle arrest was observed in DATS-treated UMSCC-22A and UMSCC-22B cells (Figure 2G,H). Interestingly, the mitotic phase arrest was not observed in both UMSCC-22A and UMSCC-22B cells pretreated with NAC (2 h), followed by DATS treatment (Figure 2I,J). Thus, the blocking of ROS generation by NAC treatment prevented DATS-induced mitotic arrest in HNSCC cells.

Further, we studied the effects of DATS on cell proliferation and survival regulatory proteins, specifically Akt and ERK; DATS treatment for 24 h decreased the phosphorylated forms of both pAkt and pERK in UMSCC-22B cells, and also caused the accumulation of mitotic marker ser10 phospho-histone H3 protein level, further suggesting the arrest of cells in the M phase of the cell cycle (Figure 2K).

These findings collectively revealed that DATS substantially increased ROS generation and indeed contributed as a trigger for DATS-induced mitotic arrest and observed molecular alterations in HNSCC cells. Since we observed a decrease in Akt and ERK1/2 signaling, the role of DATS-induced ROS was further explored in apoptosis induction in HNSCC cells.

### 3.3. DATS Induced ROS-Mediated Apoptosis in HNSCC Cells

**Figure 3:**
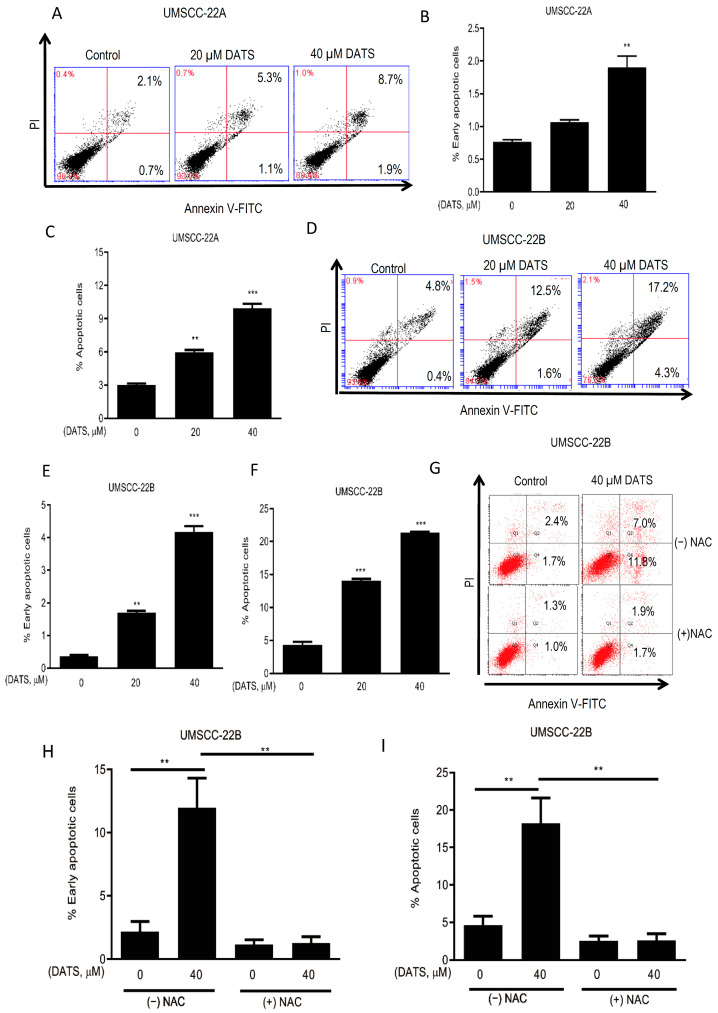
DATS-induced ROS-mediated apoptotic cell death attenuated by NAC pretreatment. Cells were treated with either DMSO or different doses of DATS for 24 h. At the end of treatments, cells were harvested and stained with annexin V and PI, and apoptotic cells were analyzed by flow cytometry. Representative histograms of UMSCC-22A (**A**) and UMSCC-22B (**D**) cells treated with DMSO (control) or 20 µM and 40 µM DATS for 24 h. Quantified data of (**B**) percent early and (**C**) total apoptotic cells of UMSCC-22A cells. Quantified data of (**E**) percent early and (**F**) total apoptotic cells of UMSCC-22B. (**G**) Representative histograms of UMSCC-22B cells treated with DMSO (control) or 40 µM DATS for 24 h in the absence or presence of NAC pretreatment. Quantified data of (**H**) percent early and (**I**) total apoptotic cells of UMSCC-22B cells. Data are shown as mean ± SEM of triplicate samples. DATS, Diallyl trisulfide, *p* < 0.001 (**), *p* < 0.0001 (***).

We wanted to study if the growth inhibitory effect of DATS occurs via apoptosis, and so apoptosis-inducing effects of DATS were assessed by Annexin V-FITC staining in UMSCC-22A and UMSCC-22B cells. DATS (20 and 40 µM) treatment induced apoptosis in a concentration-dependent manner in both UMSCC-22A and UMSCC-22B cells (Figure 3A,D). UMSCC-22A cells showed an increase in early apoptotic cells (Figure 3B) as well as a 3.3-fold (*p* < 0.001–0.0001) increase in total apoptotic cells at 24 h (Figure 3C). Similarly, UMSCC-22B cells showed an increase in early apoptotic cells (Figure 3E) and a strong increase of 4.9-fold (*p* < 0.001–0.0001) in total number of apoptotic cells following the treatment with DATS (Figure 3F). Percent live cells and percent necrotic cells upon DATS and/or NAC treatments for both the cell lines are shown in Supplementary Figure S4A–F. Further, UMSCC-22B cells were employed to study the role of ROS in DATS-induced apoptosis. Blocking of ROS generation by NAC pretreatment completely prevented DATS-induced early and late apoptosis in UMSCC-22B cells (Figure 3G–I). Thus, these findings suggested that DATS induced ROS-mediated apoptotic cell death in HNSCC cells, and that it is likely to cause DNA damage—this was investigated further.

### 3.4. DATS Induced ROS-Mediated DNA Damage and Altered the Levels of Apoptotic Regulatory Proteins

**Figure 4:**
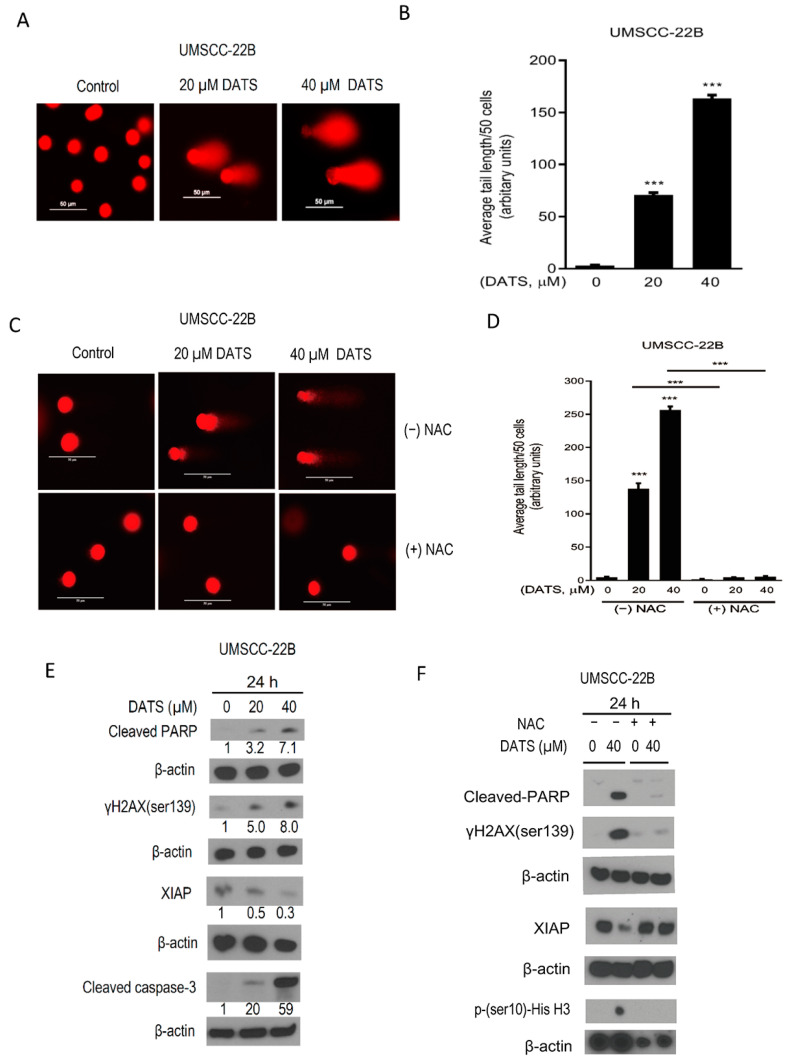
DATS induced ROS-mediated DNA damage and apoptosis in HNSCC cells and altered apoptotic regulatory proteins. (**A**) Representative images were captured using a fluorescence microscope at 200× magnification in UMSCC-22B cells (scale bars = 50 µm). (**B**) Quantification of comet tail length in UMSCC-22B cells treated with either DMSO control or 20 and 40 µM of DATS. At least 50 cells were used for statistical analysis. (**C**) Representative images were captured using a fluorescence microscope at 200× magnification in each case in the presence or absence of NAC pretreatment (scale bars = 50 µm). (**D**) quantified data of comet tail length. NAC pretreatment attenuated DATS-induced DNA damage. (**E**) Western blotting for apoptotic regulatory and DNA damage-related proteins using lysate from UMSCC-22B cells treated with DMSO control and DATS (20, 40 μM) for 24 h. (**F**) Western blotting for mitotic marker, apoptotic regulatory and DNA damage-related proteins using lysate from UMSCC-22B cells pretreated with NAC then with DMSO control and DATS (20, 40 μM) for 24 h. Data are shown as mean ± SEM of triplicate samples. DATS, Diallyl trisulfide, *p* < 0.0001 (***). The original western blot of Figure 4E,F is in Figure S6.

DATS-induced ROS-mediated potential DNA damage was investigated by performing a comet assay. The findings showed that DATS (20 and 40 µM) caused a dose-dependent significant increase 54-fold (*p* < 0.0001) in comet tails after 24 h of treatment (Figure 4A,B). Further, when the experiment was repeated with NAC-pretreated cells, no comet tails were observed, confirming the role of DATS-induced ROS in causing DNA damage in HNSCC cells (Figure 4C,D).

Next, we investigated the molecular alterations associated with DATS-induced apoptosis in HNSCC cells. The level of antiapoptotic protein X-linked inhibitor of apoptotic protein (XIAP) decreased upon DATS treatment in HNSCC cells. DATS induced an increase in levels of DNA damage marker phospho-histone H2AX (ser139), cleaved-caspase-3, and cleaved-PARP (Figure 4E). Next, we investigated if these molecular changes are also mediated through DATS-induced ROS. Cells were pretreated with NAC, which attenuated the DATS-induced reduction of antiapoptotic XIAP, and blocked the increase in phospho-histone H3, phospho-gamma-H2AX (ser139) and cleaved-PARP (Figure 4F). Similarly, DATS treatment increased the levels of apoptotic marker proteins, cleaved-PARP and cleaved caspase-3 in UMSCC-22A cells (Supplementary Figure S5). These findings suggested that DATS-caused ROS generation plays a key role in anticancer effects. DATS-induced ROS-mediated DNA damage caused apoptosis by altering the levels of apoptotic regulatory proteins in HNSCC cells.

### 3.5. DATS Treatment Decreased HNSCC Stem Cell Population

**Figure 5:**
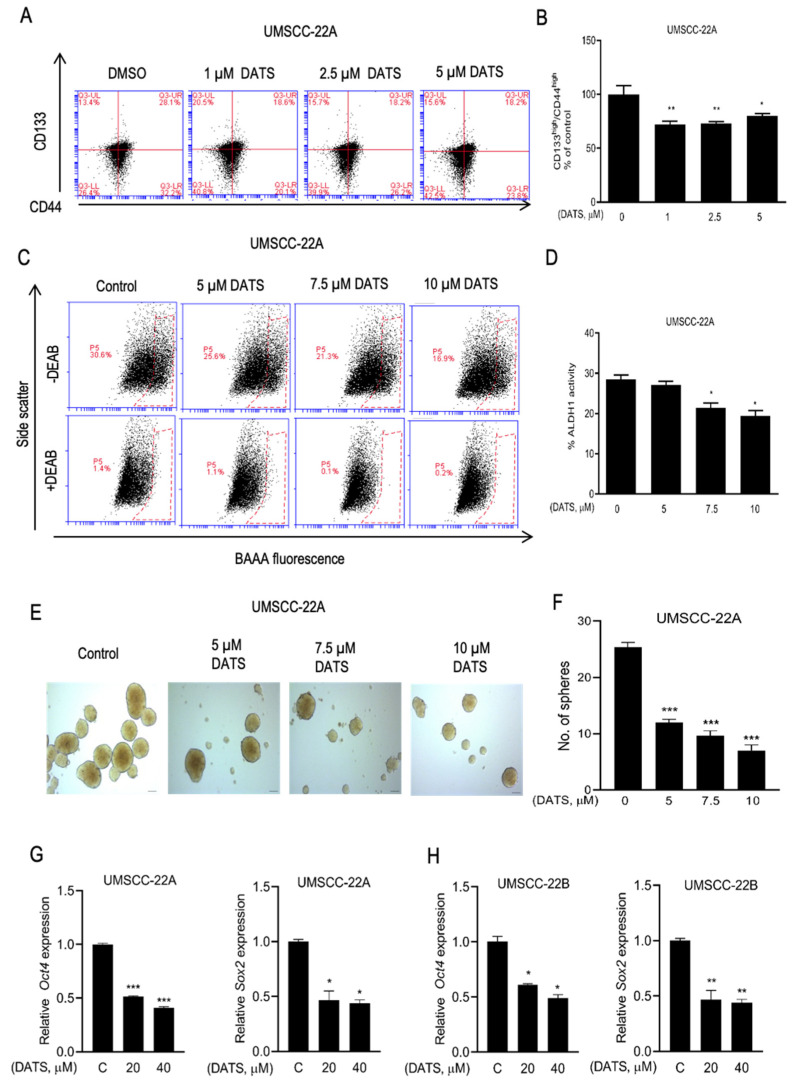
DATS decreased CSC population and stemness-related genes in HNSCC cells. (**A**) Representative histograms for CD133^high^/CD44^high^ fraction of UMSCC-22A cells after 72-h treatment with DMSO or DATS (0–5 µM). (**B**) Quantitation of CD133^high^/CD44^high^ fraction of UMSCC-22A cells after DATS treatment. (**C**) Representative flow histograms for ALDH1 activity in UMSCC-22A cells after 24-h treatment with DMSO or DATS (0–10 µM). The ALDH1 inhibitor DEAB was used as a control. (**D**) Quantitation of ALDH1 activity of UMSCC-22A cells after DATS treatment. (**E**) Representative images of spheroids resulting after 14 days of cell seeding and treatment of UMSCC-22A cells with DMSO or DATS (0–10 μM) (magnification ×100, scale bars = 200 μm). (**F**) Quantitation of number of spheroids. Cells were treated with either DMSO or 20 and 40 µM DATS for 24 h followed by quantitative analysis of stem cell-related genes *SOX2* and *Oct4* mRNA expression by real-time PCR in UMSCC-22A (**G**) and UMSCC-22B (**H**) cells relative to DMSO control. The results shown are relative to the DMSO-treated control (mean ± SEM, *n* = 3). *p* < 0.05 (*), *p* < 0.001 (**), *p* < 0.0001 (***), compared with the DMSO-treated control by one-way ANOVA followed by Dunnett’s adjustment. DATS, Diallyl trisulfide, *DEAB*, diethylaminobenzaldehyde; *BAAA*, BODIPYTM-amino acetaldehyde; C means DMSO-treated control.

DATS is reported to decrease the CSC fraction in breast cancer [26], and we checked if DATS has this effect on cancer stemness in HNSCC. Figure 5A shows representative flow histograms for CD133^high^/CD44^high^ fraction after 72 h treatment with DATS or DMSO control. The population of CD133^high^/CD44^high^ was significantly lower in DATS (1–5 µM) treated UMSCC-22A cells, compared with the DMSO control (Figure 5B). This result was confirmed by another CSC marker ALDH1 activity assay. As anticipated, ALDH1 activity was reduced in the presence of DATS (5–10 µM), compared to the DMSO control in UMSCC-22A cells after 24 h of treatment (Figure 5C,D). Further, the effect of DATS on the self-renewal ability of head and neck cancer stem cells was studied by sphere formation assay. Figure 5E depicts the representative spheroids resulting after 14 days of seeding in the absence of the presence of DATS. DATS (5–10 µM) decreased the sphere-forming ability, both significantly and dose-dependently (52–72%, *p* < 0.001) in UMSCC-22A cells, compared with the DMSO control (Figure 5F). Further, DATS treatment for 24 h downregulated the expression of stemness-related genes, including *Oct4* and *SOX-2*, in both UMSCC-22A (Figure 5G) and UMSCC-22B (Figure 5H) cells.

### 3.6. DATS Treatment Inhibited HNSCC Xenograft Growth as Well as CSC Fraction In Vivo

**Figure 6:**
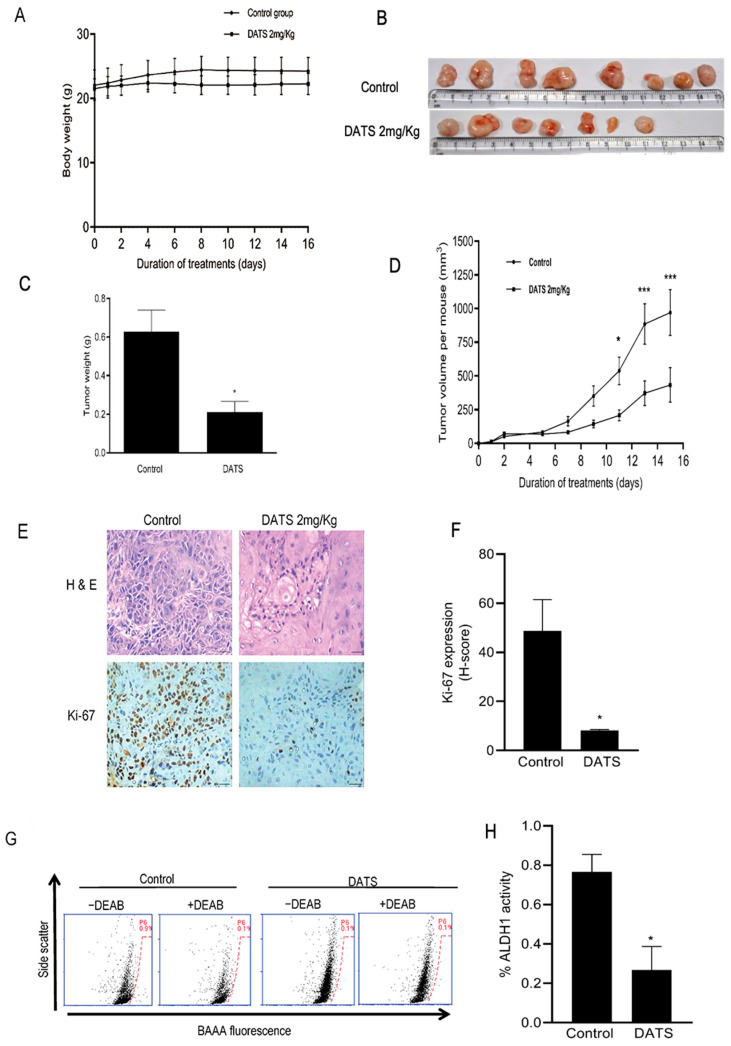
DATS administration inhibited the tumor growth of UMSCC-22B cells and cancer stem cells in vivo. (**A**) Body weights for control mice and those treated with DATS. (**B**) Representative tumor images of control mice and DATS-treated mice. (**C**) Average tumor weight in control and DATS-treated mice. In one mouse of the DATS group, the tumor regressed drastically on one side. (**D**) Average tumor volume as a function of time in control mice and DATS-treated mice (oral gavage administration, five times per week). There were four mice each in the control and DATS treatment group with tumor cells implanted on both the left and right flank of each mouse. (**E**) Representative images of Ki-67 immunohistochemical staining from control mouse tumor and DATS-treated mouse tumor (magnification ×200, scale bar = 100 μm). (**F**) Quantification of Ki-67 protein expression. The result shown is the mean H-score (*n* = 3 for control, and *n* = 3 for the DATS-treated group). (**G**) Representative flow histograms for ALDH1 activity from single cells isolated from control and DATS-treated mice tumors. The ALDH1 inhibitor DEAB was used as a control. (**H**) Quantitation of ALDH1 activity of respective groups. The results shown are mean ± SEM. The *p*-value was calculated by a two-sided Student’s *t*-test. DATS, Diallyl trisulfide, *p* < 0.05 (*), *p* < 0.0001 (***).

After referring to in vitro findings, we further checked the in vivo efficacy of DATS for the inhibition of head and neck cancer stem cells by using a UMSCC-22B xenograft model. The body weight gain of mice did not differ in DATS (2 mg/Kg body weight) and DMSO control-treated groups during 15 days of the treatment (Figure 6A). The tumor incidence was 100% in vehicle-treated control mice, but was reduced in the DATS treatment group, where only 7 out of 8 xenografts grew to form tumors (Figure 6B). Average tumor weight was lower (66%, *p* < 0.05) in the DATS-treated group, compared to the control group (Figure 6C). DATS also significantly decreased the kinetics of tumor growth, showing a 55% (*p* < 0.0001) decrease in tumor volume during 15 days of the experiment (Figure 6D). In the immunohistochemical analysis of tumors, DATS showed a strong decrease in Ki-67-positive cells (83%, *p* < 0.05) (Figure 6E,F). In order to assess the effect of DATS on CSC in tumors, it was analyzed for ALDH1 activity: Figure 6G shows flow histograms for ALDH1 activity in tumor cells from the control and DATS treatment groups. The ALDH1 activity reduced by 3.5-fold (*p* < 0.05) in tumors treated with DATS, compared to the vehicle-treated control (Figure 6H). Overall, these findings suggested that DATS has strong efficacy in inhibiting HNSCC tumor growth, as well as the CSC fraction in the tumors.

## 4. Discussion

The existing chemotherapeutic counter-HNSCC agents are non-selective and associated with toxicities as well as the emergence of chemoresistance [32], so new agents that can safely be integrated into current treatment regimens to improve overall therapeutic outcomes are desired. The development of agents that are nontoxic to normal cells but can inhibit the growth and survival of HNSCC and cancer stem cells could have a significant impact on disease in reducing the cost of treatment, morbidity, and mortality in HNSCC patients [2]. Epidemiological studies and population-based case-control studies revealed the health benefits of Allium vegetables, whose therapeutic benefits are attributed to organosulfur compounds (OSCs), diallyl sulfide (DAS), diallyl disulfide (DADS), and diallyl trisulfide (DATS) [33]. DATS is the most effective OSC found in garlic and has been shown to conduct anticancer activities against prostate cancer [34,35], breast cancer [25,36], and lung cancer [37,38]. The present study established its anticancer activity against HNSCC.

The inhibition of cell cycle progression is known as a mechanism to suppress the growth and proliferation of cancer cells. DATS strongly inhibited the growth and proliferation of HNSCC cells in a concentration- and time-dependent manner, and so its effect on cell cycle progression was anticipated. DATS showed G2-M phase cell cycle arrest with the accumulation of cyclin B1 in HNSCC cells. In cycling cells, the synthesis of cyclin B1 increases abruptly during the late S phase to the early G2 phase, and its level peaks during the metaphase and anaphase transition and then decreases upon completion of mitosis [39]. Degradation of cyclin B1 is essential for mitotic exit and cytokinesis [40]. Since DATS increased the levels of cyclin B1 protein, we hypothesized that DATS-treated UMSCC-22A and UMSCC-22B cells would be unable to exit the mitosis phase, on the grounds that a similar phenomenon was reported in prostate cancer cells [21]. The DATS-induced mitotic arrest was confirmed by flow cytometric analysis of Ser-10 phosphorylation of histone H3, a sensitive marker for mitotic cells that is essential for regulating chromatin decondensation and protein-protein interactions. N-terminus Ser-10 phosphorylation of histone H3 begins in prophase, and reaches a peak in metaphase and decreases during anaphase. Thus, agents triggering premature chromosome condensation have been shown to increase Ser-10 phosphorylation of histone H3 [21]. DATS treatment decreased the levels of CDK1 and increased the levels of universal cell cycle inhibitor Cip1/p21 protein, as earlier reported in studies of prostate cancer [34] and gastric cancer [41]. Further, DATS treatment decreased the phosphorylation of Akt and ERK1/2 proteins, whose signaling pathways are crucial mediators of cell proliferation, survival, and metastasis [42,43]. These results together revealed that DATS inhibited mitogenic and survival signaling, and induced mitotic phase arrest in cell cycle progression, leading to growth arrest of HNSCC cells.

Apoptosis is a complex and highly regulated process involving proapoptotic proteins like caspases and Bax, antiapoptotic regulatory proteins such as Bcl-2, and inhibitors of apoptotic (IAP) proteins like the X-linked inhibitor of apoptotic protein (XIAP). The IAP family proteins inhibit apoptosis by binding to and inhibiting the activation of caspases [35]. DATS-induced cell death via apoptosis in HNSCC cells was accompanied by an increase in apoptotic markers cleaved-PARP and cleaved-caspase-3 and a decrease in anti-apoptotic protein XIAP. Next, the triggering mechanism of DATS-induced apoptosis was investigated.

Reactive oxygen species (ROS) are byproducts of metabolism and xenobiotic exposure and, depending on their concentration, can be beneficial or detrimental to cells. ROS function as redox messengers in intracellular signaling and regulate many biological processes at physiologically low levels, whereas excess ROS is known to induce cell death [44]. DATS was found to induce a significant level of DNA damage in HNSCC cells, which was evident from the comet assay. Therefore, we hypothesized that ROS mediates the effects of DATS in inducing mitotic arrest and apoptosis. To prove this hypothesis, we utilized NAC pretreatment to cells, which attenuated the DATS-induced mitotic arrest, apoptosis and DNA damage in HNSCC cells. This finding suggested that ROS acts as an upstream signaling molecule for the DATS-mediated DNA damage, cell cycle arrest, and apoptosis in these cancer cells.

Despite the progress in the understanding of the biology of CSC development, the direct therapeutic targeting of these cells remains challenging. CSC-targeting agents alone may not be able to eradicate the tumors due to the possible conversion of non-CSC into CSC. Studies have shown that phytochemicals like benzyl isothiocyanate [45,46,47], sulforaphane [48], and withaferin A [49] suppressed the growth of CSC, along with bulk cancer cells. Recently, Kim et al. reported that DATS treatment decreased CSC fraction in breast cancer, both in vitro and in vivo [26,36], and on this basis we anticipated the effects of DATS on HNSCC stem cells. DATS treatment significantly lowered the CD133^high^/CD44^high^ fraction, ALDH1 activity and spheroid formation, and the inhibition of CSC was evident at non-cytotoxic doses of DATS. Nevertheless, the DATS concentrations required to inhibit HNSCC CSC are physiologically achievable, since the maximum blood concentration of 31 µM in rats has been achieved after a single intravenous injection of 10 mg of DATS [50]. DATS treatment downregulated the stemness-related genes *Oct4* and *SOX2*. DATS could be inhibiting the cancer stemness of HNSCC cells by decreasing the expression of stemness-related genes *Oct4* and *SOX2*. In in vivo study, DATS treatment reduced ALDH1 activity by 3.5-fold in tumor cells from DATS-treated mice. These findings suggested that DATS inhibits HNSCC CSC, both in vitro and in vivo. Further, it was also observed that DATS treatment reduced tumor weight and tumor volume in the UMSCC-22B xenograft study. Immunohistochemical analysis of tumor sections showed a decrease in the Ki-67 proliferation marker in tumors of the DATS-treated group of mice. Together, these findings suggested that DATS has strong potential to target both bulk as well as cancer stem cells in HNSCC, leading to suppression of tumor growth.

Several studies, both by ourselves and others, have, in considering the toxicity evaluation of DATS for its potential human uses, reported its non-toxicity on non-cancer cell lines. The similar DATS concentrations (20–40 µM) did not affect the viability of normal prostate epithelial cells (PrEC cells) mammary epithelial cell lines (MCF-10A and MCF-12A cells), and it was also found that DATS treatment did not disrupt actin cytoskeleton in MCF-10A cells [31,34,51,52], nor cell cycle progression and cell growth in normal cells. In the present study, DATS treatment was relatively better tolerated in human embryonic kidney cells. DATS treatment did not induce reactive oxygen species generation at 40 µM in PrEC cells, and these cells showed more resistance to apoptosis, even at 160 µM concentration, as compared to cancer cells [53]. DATS also acts as a potent immunological adjuvant in mice, and its application provides an effective strategy to improve the efficacy of immune responses in vivo against cancer [54]. Furthermore, in a human clinical trial with 200 mg of synthetic DATS (allitridum) administered every day for one month per year for three years in combination with selenium, it was found to be well tolerated in gastric cancer patients [55]. Such studies support the potential use of DATS in humans.

## 5. Conclusions

**Figure 7:**
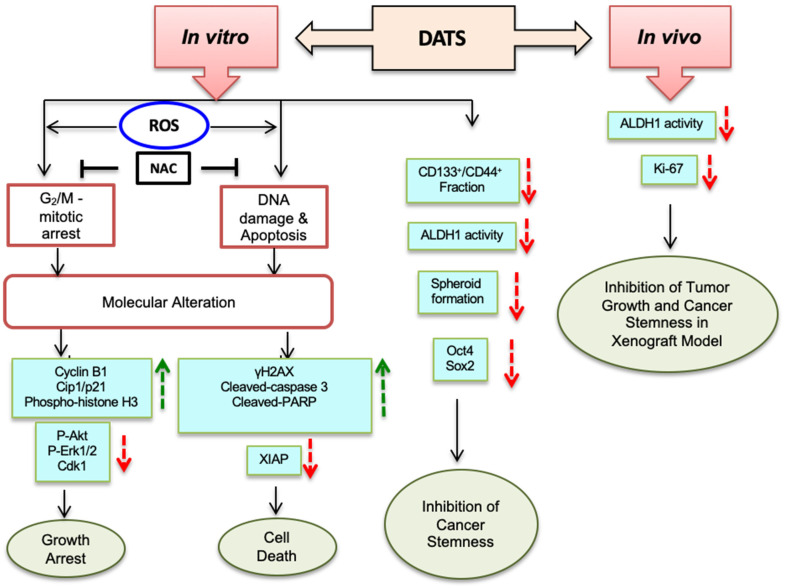
Mechanism of DATS-induced cell cycle arrest, apoptosis, DNA damage, and inhibition of cancer stemness. DATS-induced ROS-mediated mitotic arrest, apoptosis, and DNA damage in HNSCC cells by altering the levels of the cell cycle regulatory proteins, apoptotic regulatory proteins, and DNA damage marker γ-H2AX. NAC (N-acetyl cysteine) pretreatment attenuates DATS-induced ROS-mediated mitotic arrest, apoptosis, and DNA damage. DATS treatment inhibited the cancer stem cell (CSC) fraction in vitro. DATS treatment inhibited the growth of HNSCC tumor xenograft and CSC fraction in vivo. Red arrow indicates decrease and green arrow indicates increase.

This study established the anticancer activity of DATS against HNSCC. DATS inhibited cell proliferation and induced ROS-mediated DNA damage, mitotic arrest, and apoptosis in HNSCC cells in vitro, and also inhibited tumor growth in vivo. Further, DATS inhibited HNSCC stem cells, both in vitro and in vivo (Figure 7). Therefore, the anticancer effects of garlic constituent DATS could be further explored, with the aim of gaining improved insight into its clinical efficacy against HNSCC.

## Data Availability

Data can be shared upon reasonable request.

## Supplementary Materials

The following supporting information can be downloaded at: https://www.mdpi.com/article/10.3390/cancers16020378/s1, Figure S1: DATS treatment moderately inhibited cell viability in HEK 293 cells. Figure S2: DATS treatment altered cell cycle regulatory proteins in HNSCC cells. Figure S3: DATS treatment caused ROS generation in HNSCC cells. Figure S4: DATS induced apoptotic cell death in HNSCC cells. Figure S5: DATS treatment altered apoptotic regulatory proteins inHNSCC cells. Figure S6: Original western blots.
