# Supplementary material for: Diallyl Trisulfide Induces ROS-Mediated Mitotic Arrest and Apoptosis and Inhibits HNSCC Tumor Growth and Cancer Stemness
Source: Cancers (Basel). 2024 Jan 16;16(2):378. doi: 10.3390/cancers16020378 (PMC10814060; doi:10.3390/cancers16020378)

Figure 1I

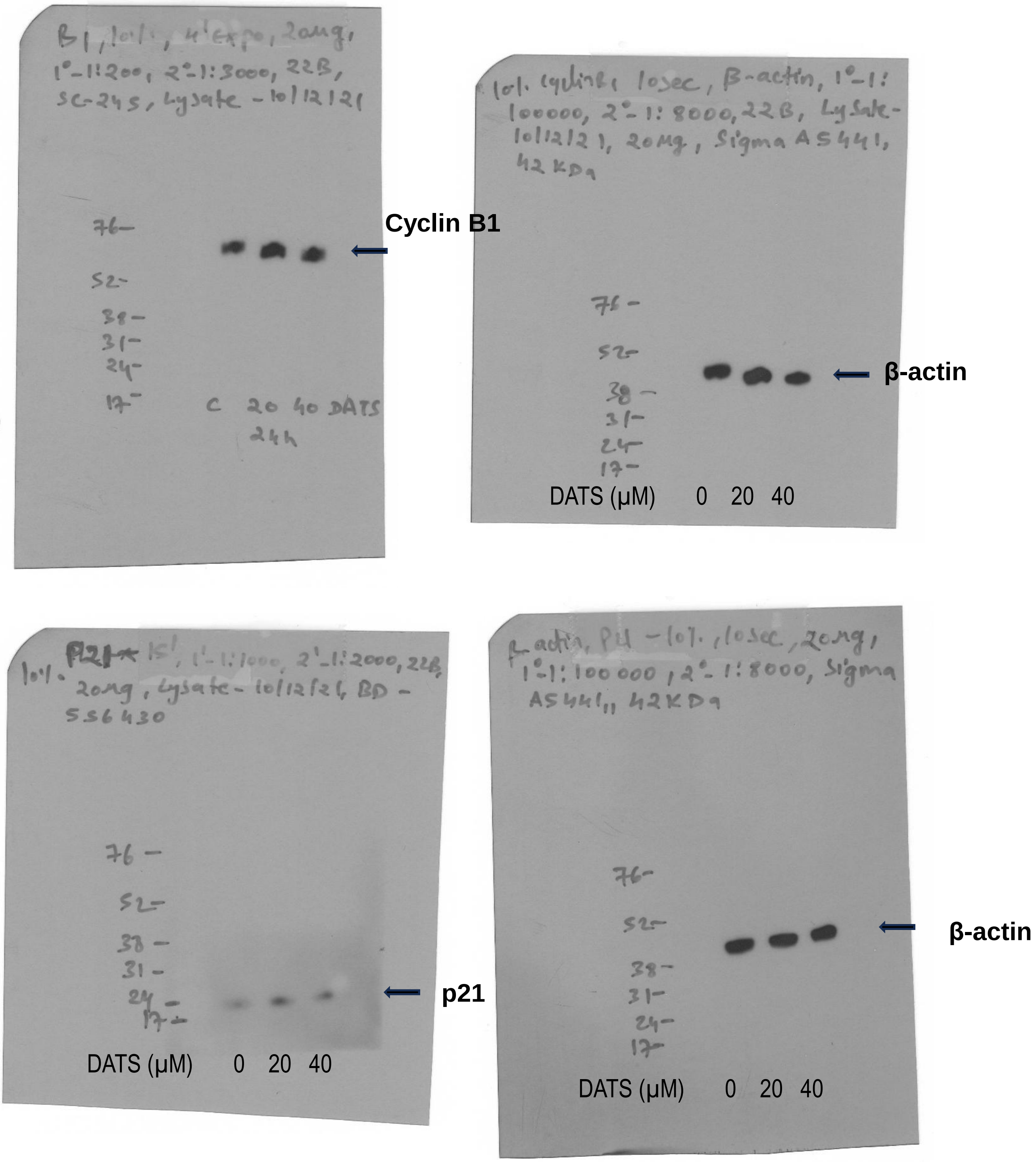

Cyclin B1
β-actin
DATS (μM) 0 20 40
p21
DATS (μM) 0 20 40
β-actin
DATS (μM) 0 20 40

Figure 1I

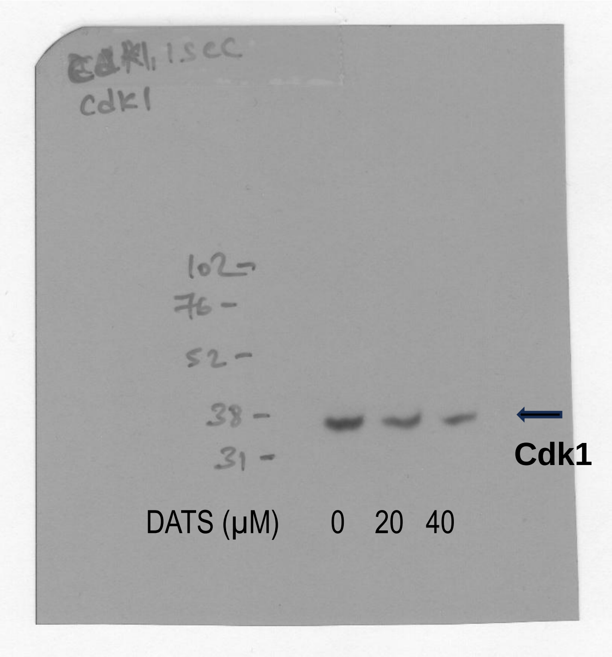

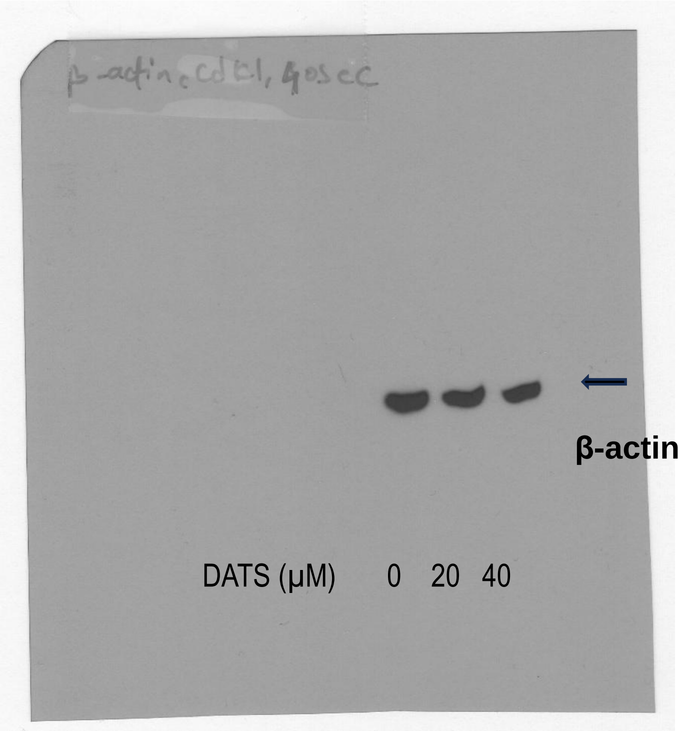

Figure 2K

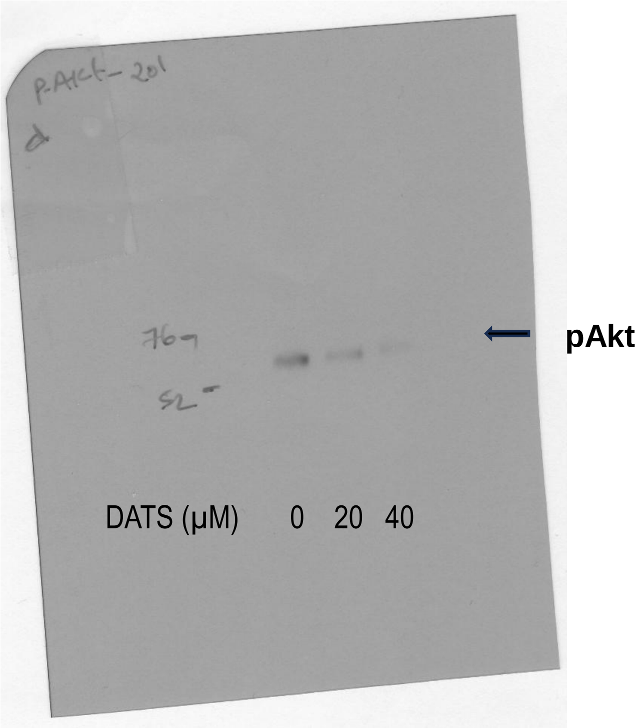
pAkt
76
52
DATS (µM) 0 20 40

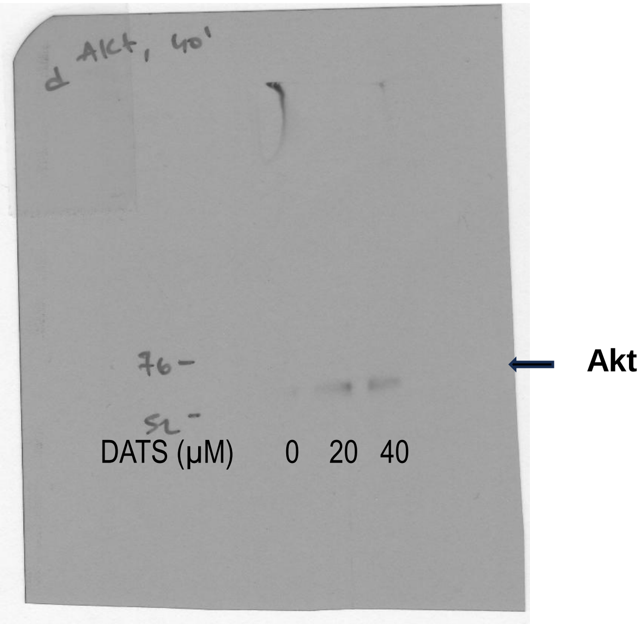
Akt
76
52
DATS (µM) 0 20 40

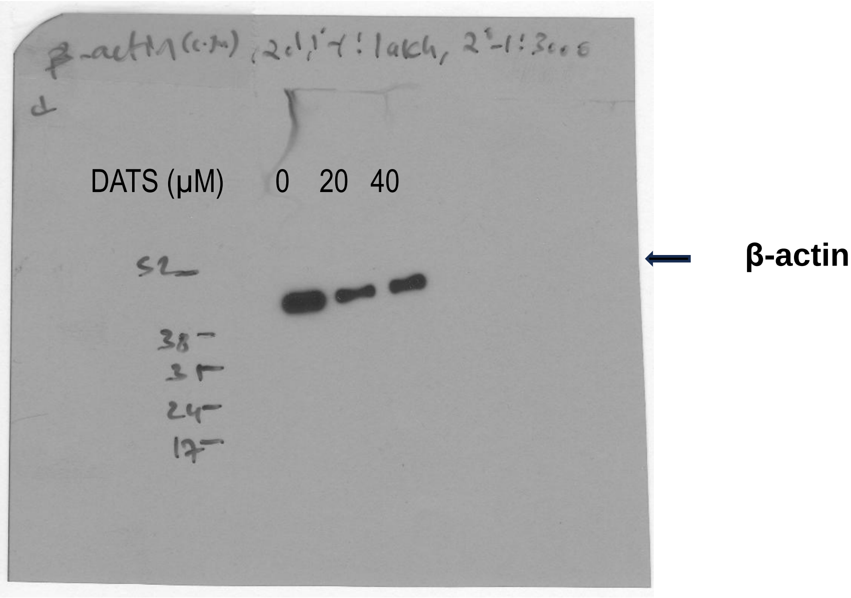
DATS (µM) 0 20 40
β-actin
52
38
31
24
17

Figure 2K

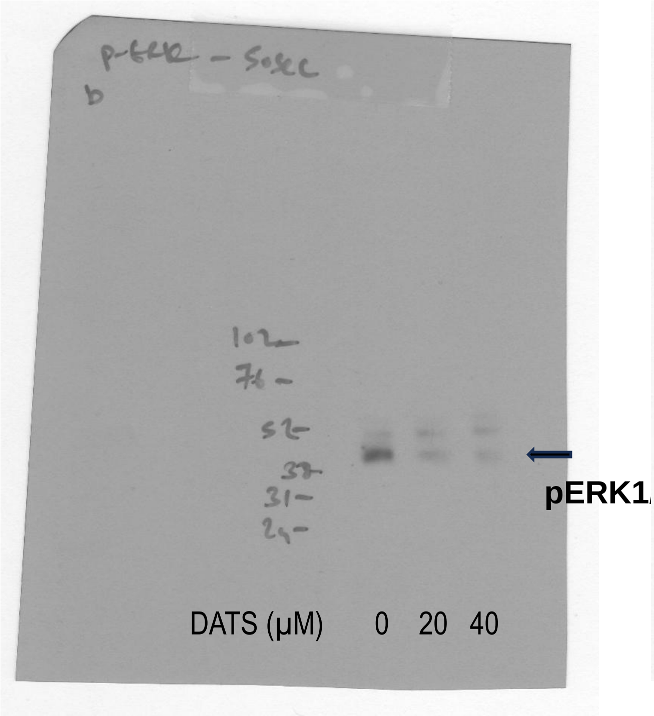

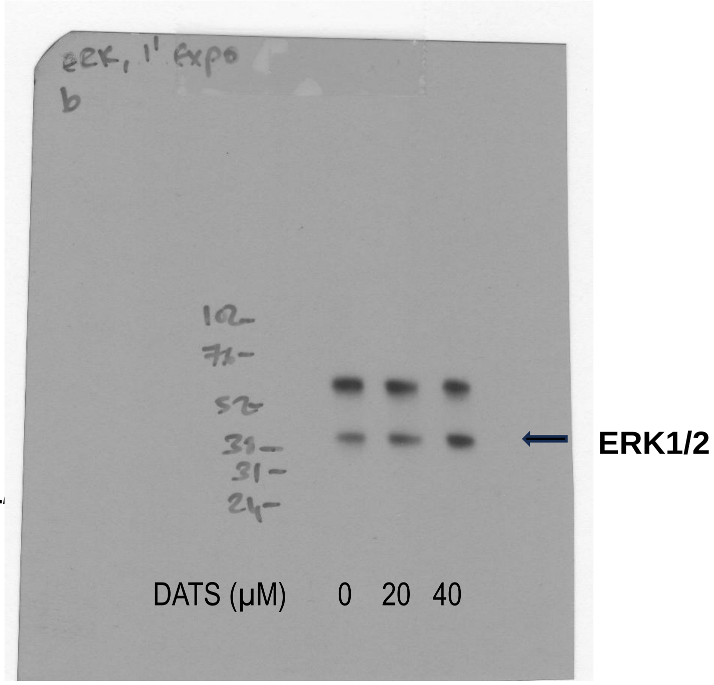

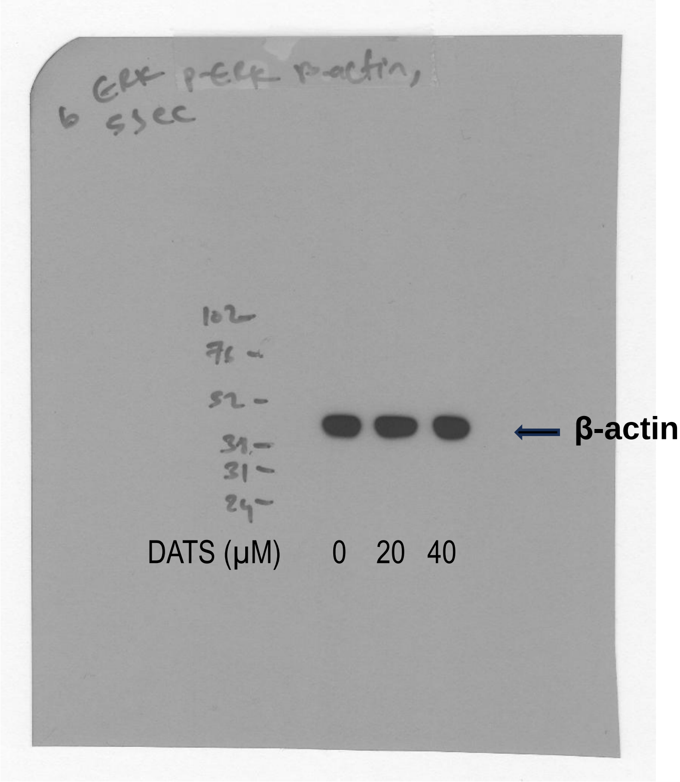

Figure 2K

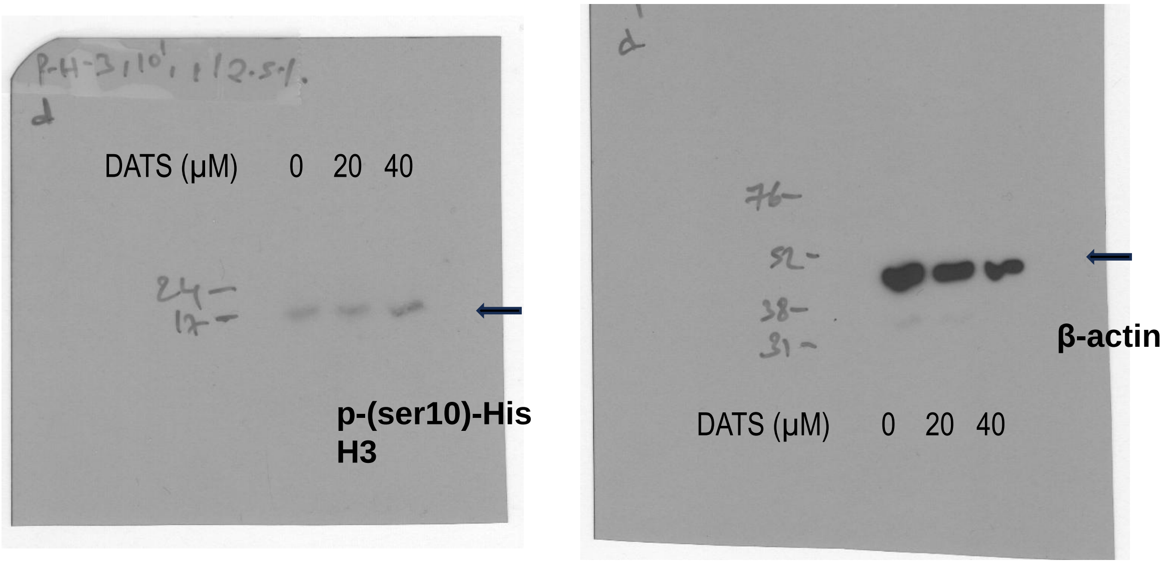

Figure 4E

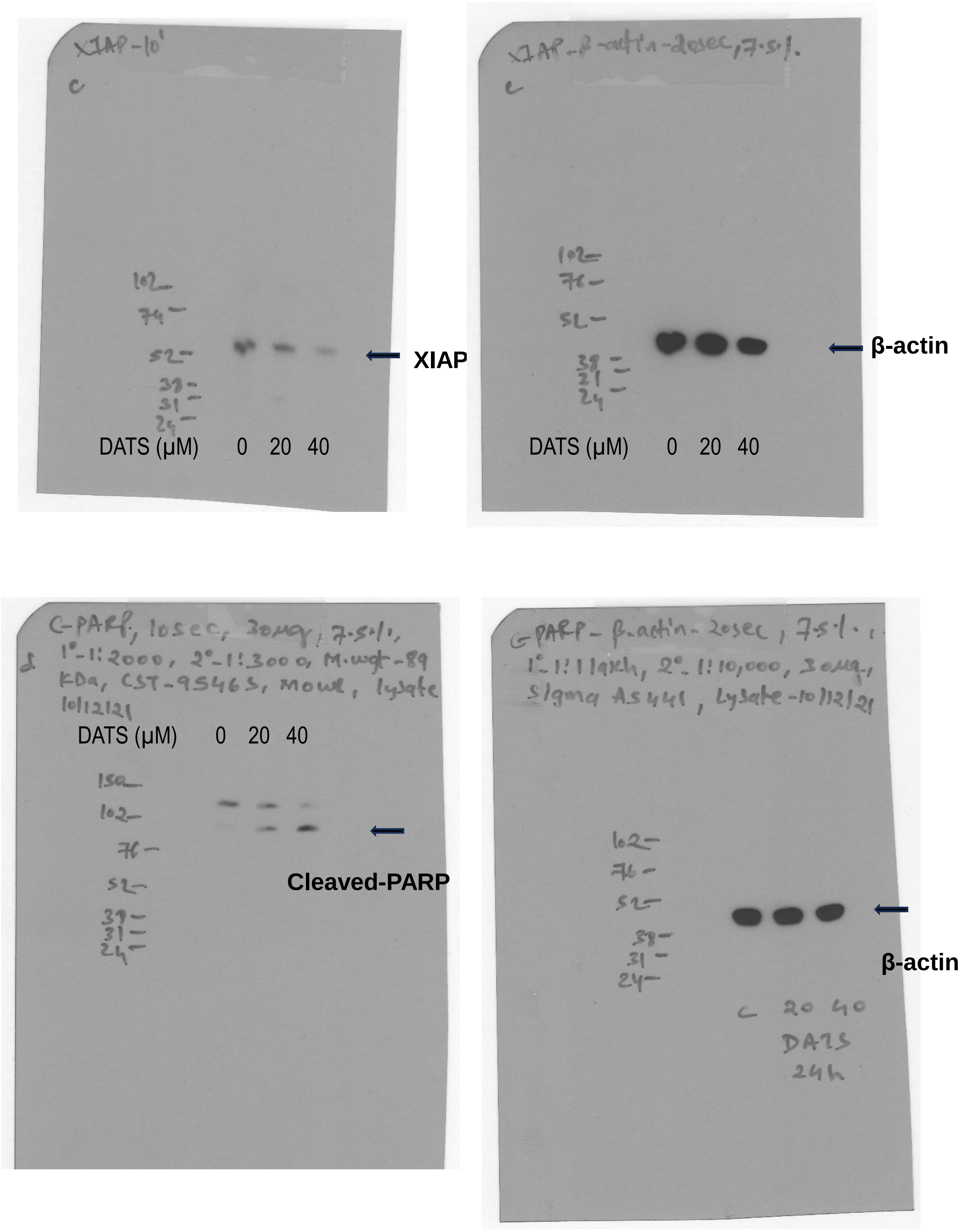
XIAP
DATS (μM) 0 20 40
β-actin
DATS (μM) 0 20 40
DATS (μM) 0 20 40
Cleaved-PARP
β-actin

Figure 4E

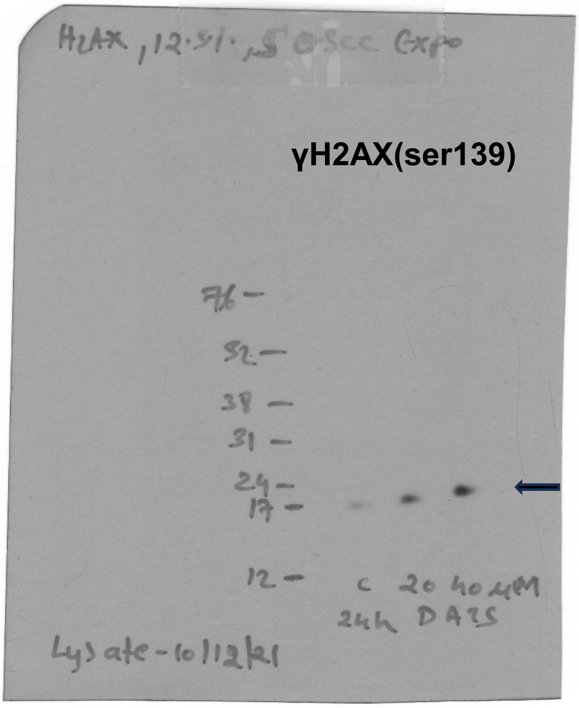

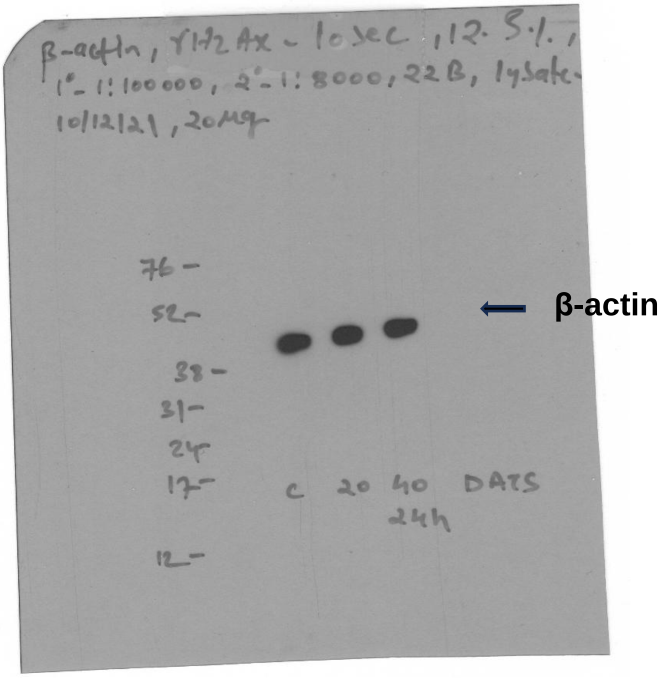

β-actin

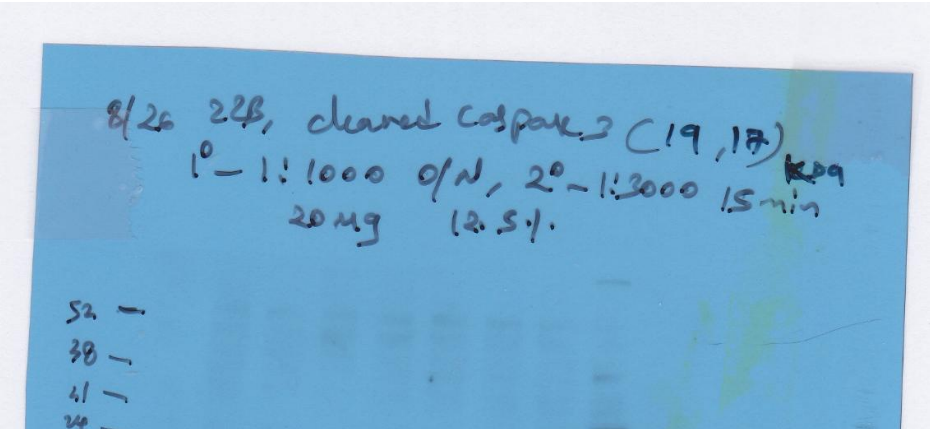

Cleaved caspase-3

DATS (μM) 0 20 40

24h

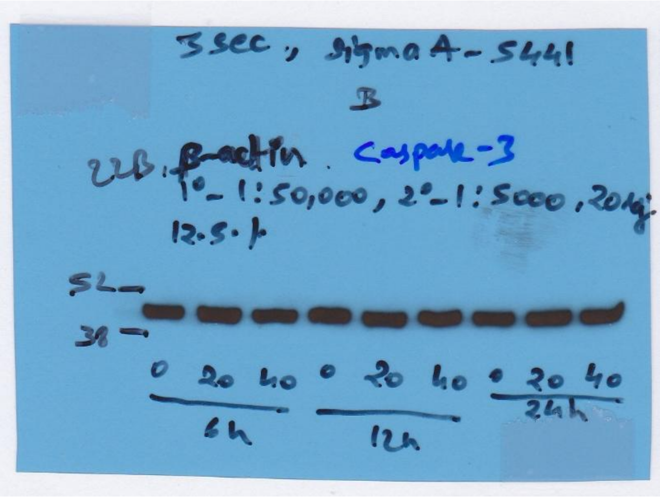

β-actin

Figure 4F

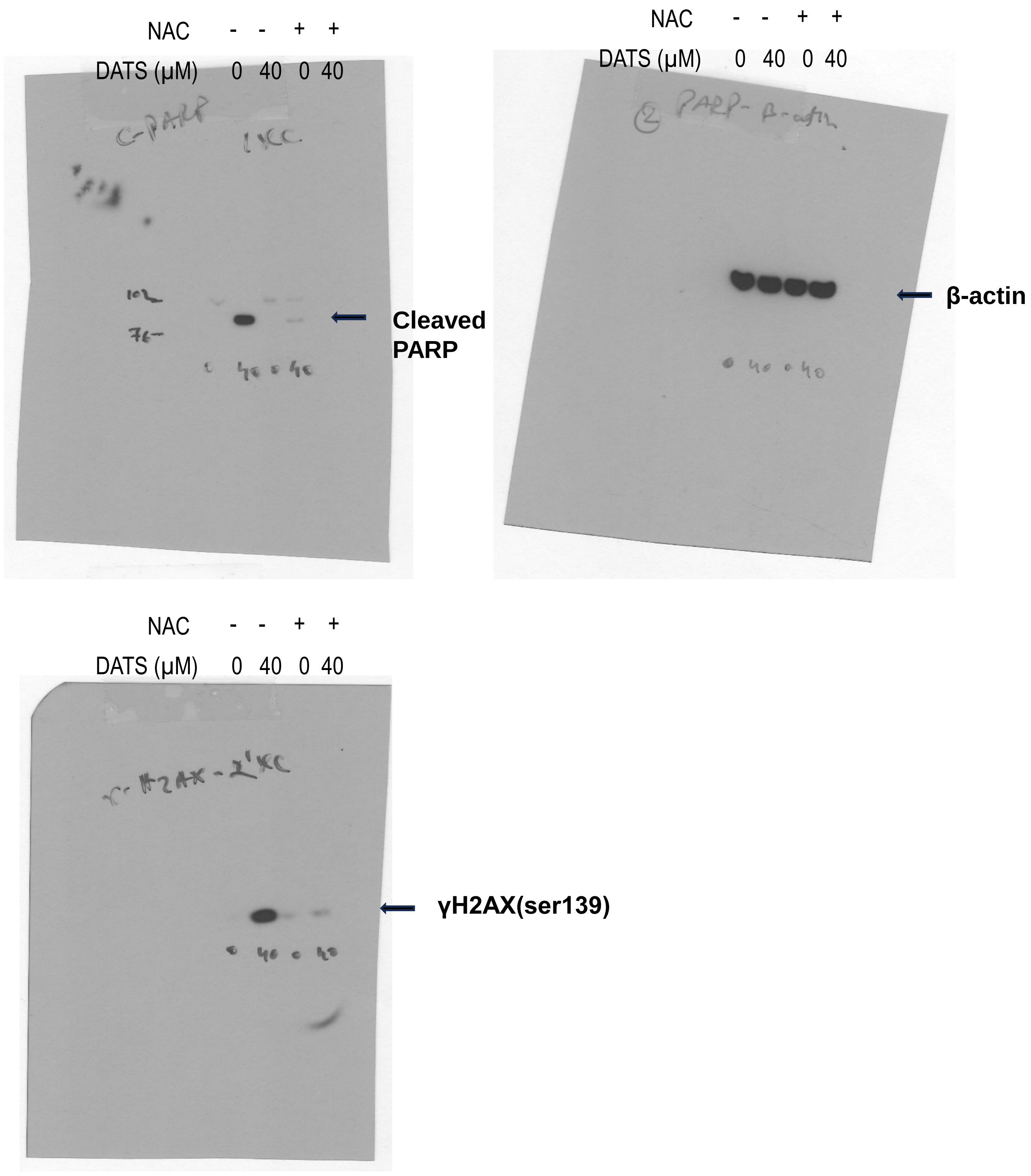

NAC - - + +
DATS (μM) 0 40 0 40
Cleaved PARP
NAC - - + +
DATS (μM) 0 40 0 40
β-actin
NAC - - + +
DATS (μM) 0 40 0 40
γH2AX(ser139)

Figure 4F

NAC - - + +

DATS (μM) 0 40 0 40

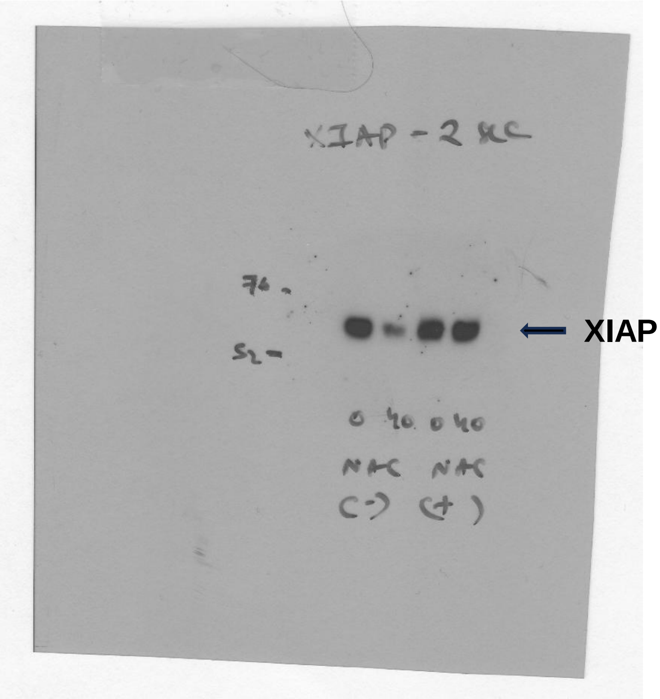

XIAP

NAC - - + +

DATS (μM) 0 40 0 40

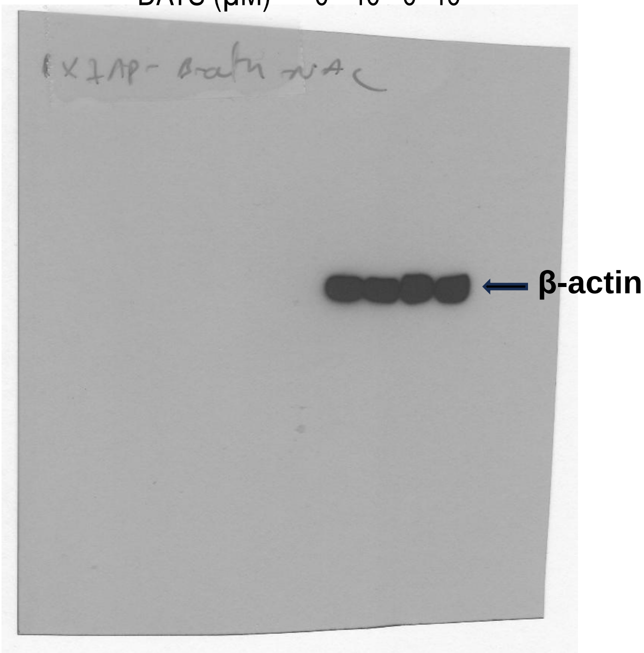

β-actin

NAC - - + +

DATS (μM) 0 40 0 40

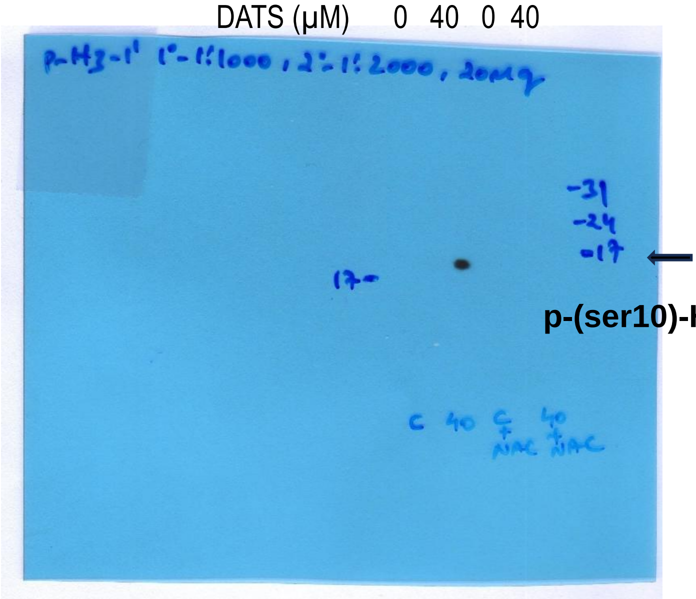

p-(ser10)-His

NAC - - + +

DATS (μM) 0 40 0 40

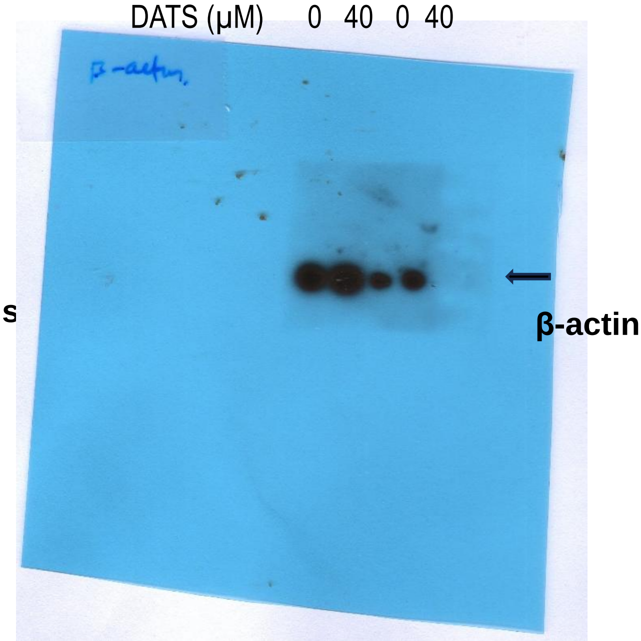

β-actin

Supplementary Figure S2

Cyclin B1

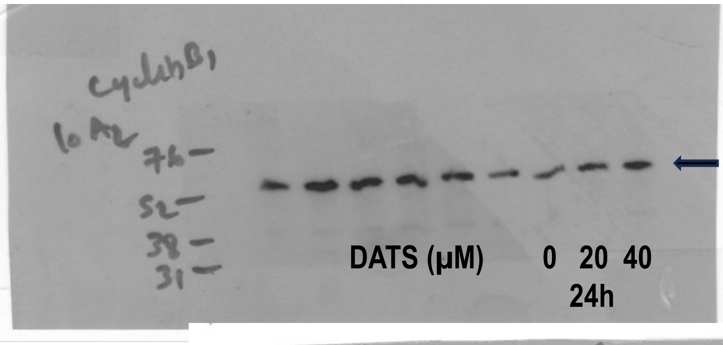

Cyclin B1

β- actin

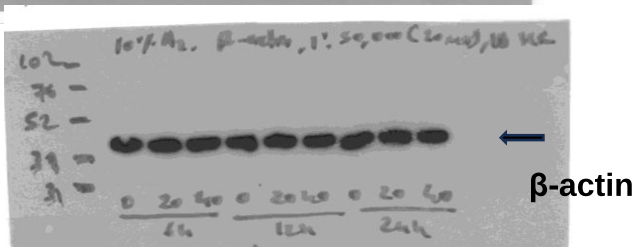

β-actin

DATS (μM) 0 20 40
24h

p21

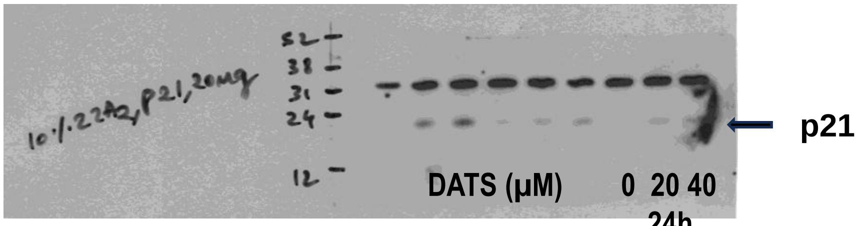

p21

β- actin

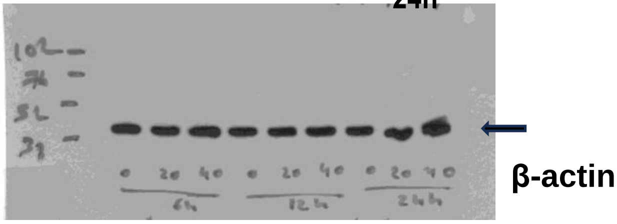

β-actin

DATS (μM) 0 20 40
24h

Supplementary Figure S5

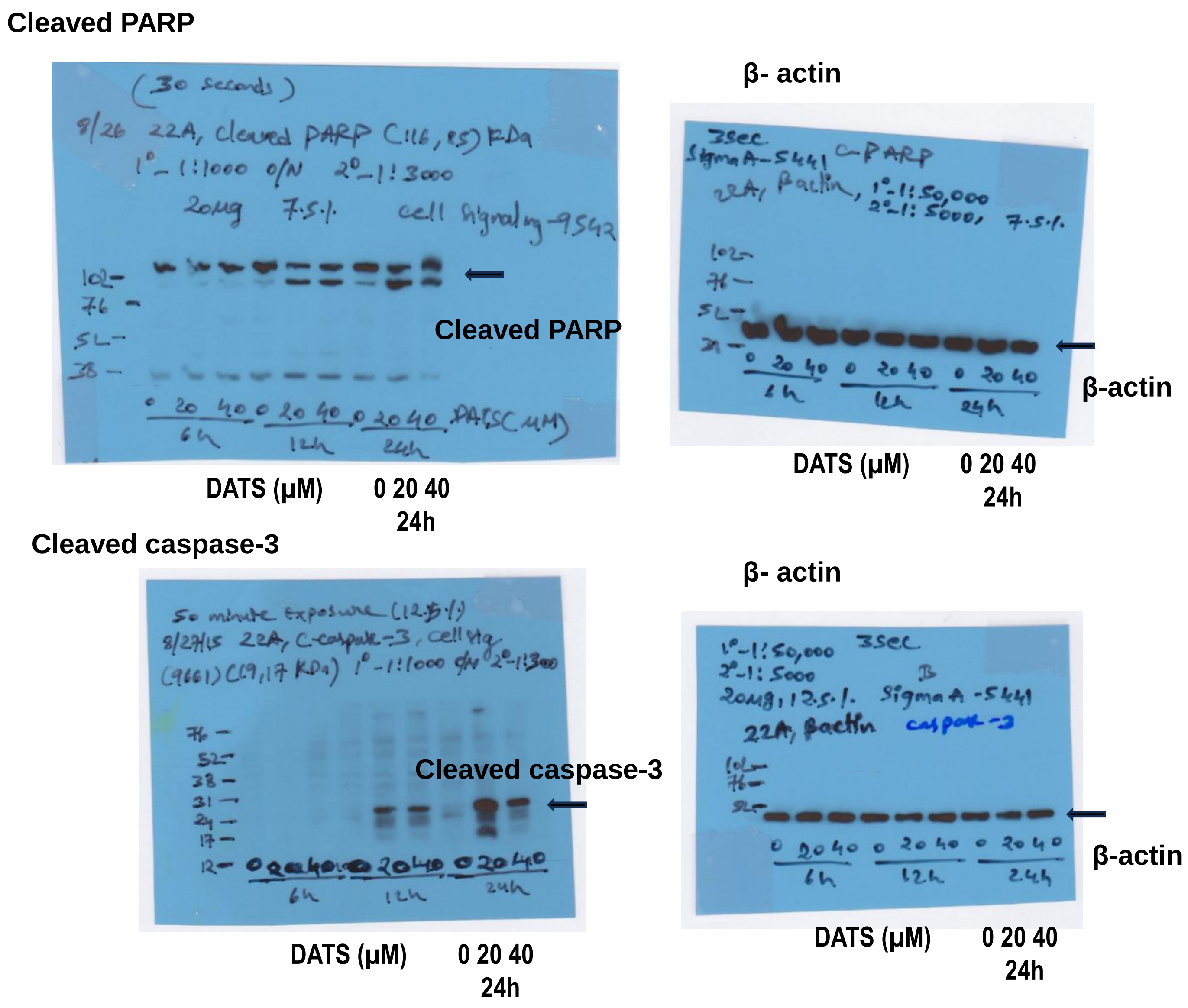

Supplement: Supplementary file 1 [file cancers-16-00378-s001.zip › cancers-2746606 -Figure S6 (Original Western scanned blots).pdf]
