# Supplementary material for: Diallyl Trisulfide Induces ROS-Mediated Mitotic Arrest and Apoptosis and Inhibits HNSCC Tumor Growth and Cancer Stemness
Source: Cancers (Basel). 2024 Jan 16;16(2):378. doi: 10.3390/cancers16020378 (PMC10814060; doi:10.3390/cancers16020378)
Supplement: Supplementary file 1 [file cancers-16-00378-s001.zip › cancers-2746606 -Figures S1–S5.pdf]

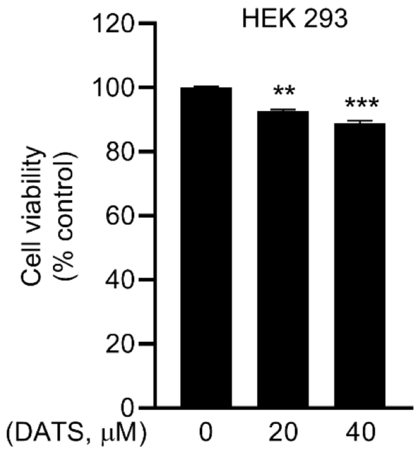

Supplementary Figure S1. DATS treatment moderately inhibited cell viability in HEK 293 cells. Cells were treated with vehicle (DMSO) alone or 20-40 µM of DATS in a fresh medium. After 24 h of these treatments, viable cells were counted using trypan blue staining and hemocytometer. **p<0.01, ***p<0.001

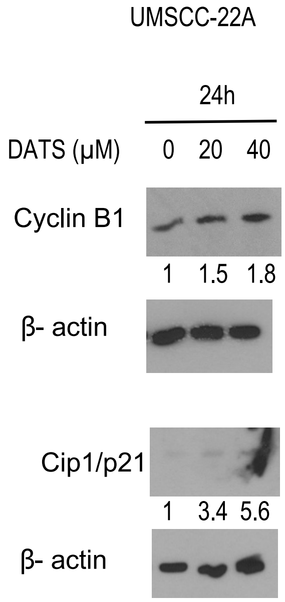

Supplementary Figure S2: DATS treatment altered cell cycle regulatory proteins in HNSCC cells. Western blotting for cell cycle proteins using lysate from UMSCC-22A cells treated with DMSO control and DATS (20 and 40 µM) for 24h. Number below each band represents the densitometric data of fold change to that of control.

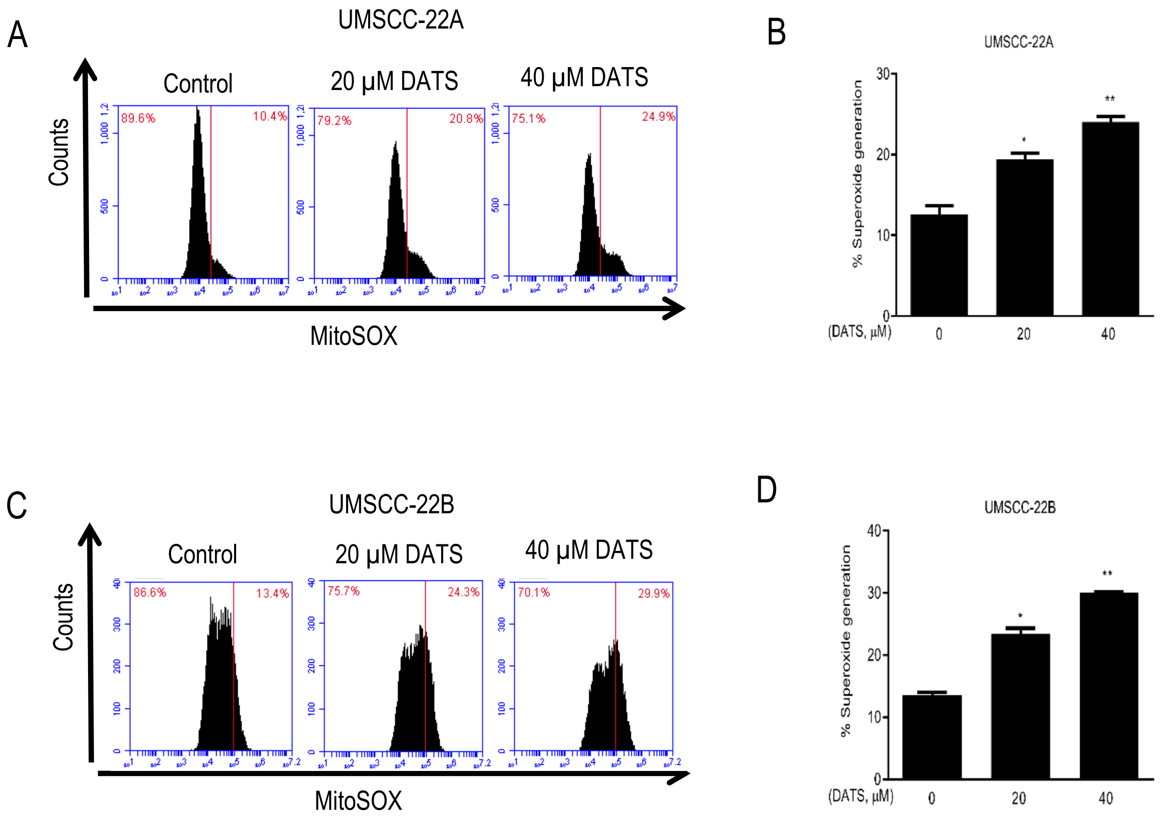

Supplementary Figure S3. DATS treatment caused ROS generation in HNSCC cells. Representative histograms of (A) UMSCC-22A and (C) UMSCC-22B cells treated with different doses of DATS (20, 40μM) for 3h and analyzed by flow cytometry for MitoSOX Red fluorescence in HNSCC cells. DATS treatment caused superoxide generation in HNSCC cells and quantitation of superoxide generation in (B) UMSCC-22A and (D) UMSCC-22B cells compared to DMSO control. *p<0.05, **p<0.01.

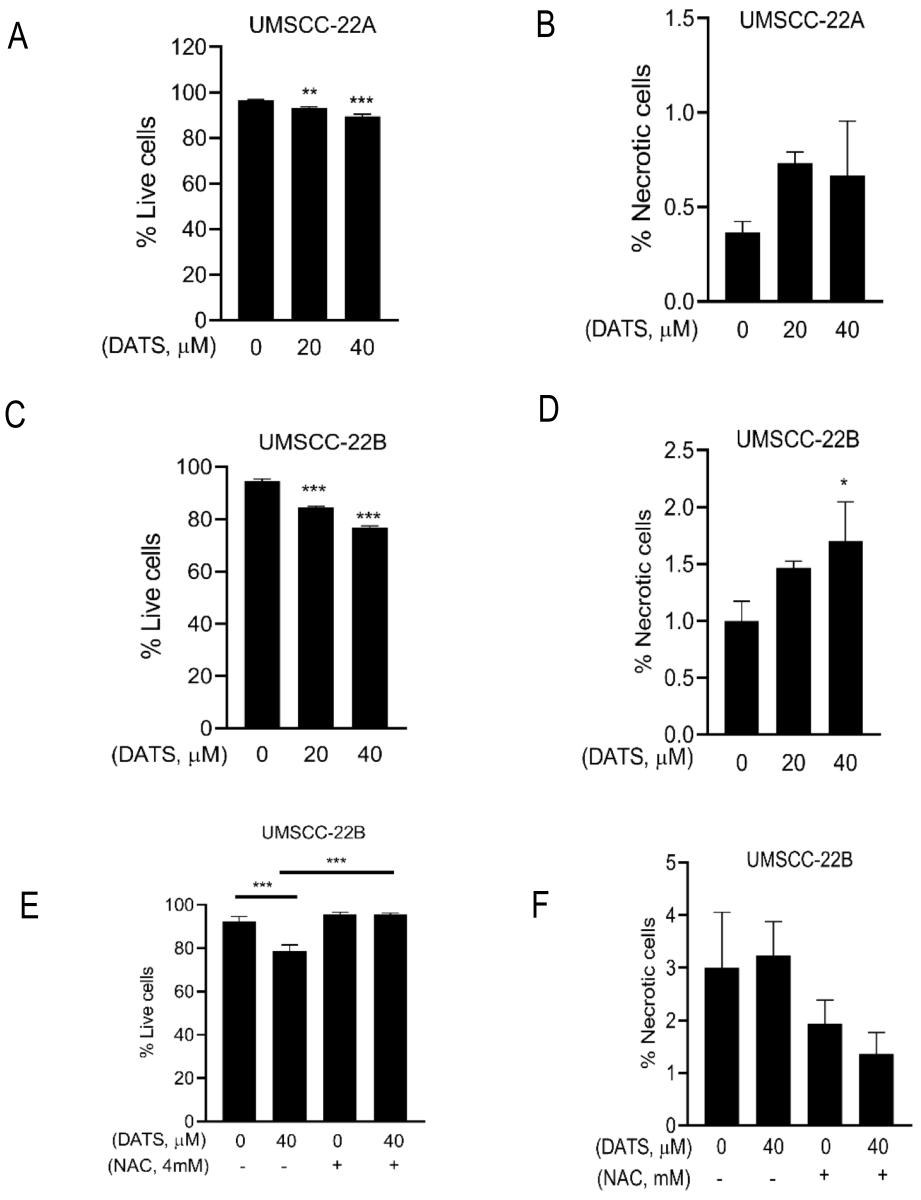

Supplementary Figure S4. DATS induced apoptotic cell death in HNSCC cells: UMSCC-22A and UMSCC-22B cells treated with DMSO (control) or 20 µM and 40 µM DATS for 24h. Quantitative data of (A) percent live and (B) percent necrotic cells of UMSCC-22A cells. Quantitative data of (C) percent live and (D) percent necrotic cells of UMSCC-22B cells. Quantitative data of (E) percent live and (F) percent necrotic cells of UMSCC-22B cells treated with DMSO (control) or 40 µM DATS for 24h in the absence or presence of 4 mM NAC (N-acetyl cysteine) pretreatment. *p<0.05, **p<0.01, ***p<0.001

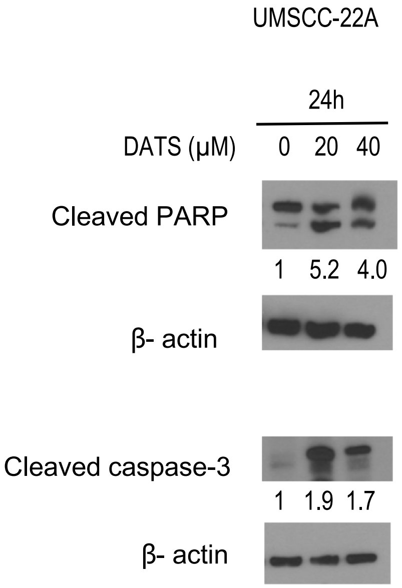

Supplementary Figure S5: DATS treatment altered apoptotic regulatory proteins in HNSCC cells. Western blotting for apoptotic regulatory proteins using lysate from UMSCC-22A cells treated with DMSO control and DATS (20 and 40 μM) for 24h. Number below each band represents the densitometric data of fold change to that of control.
